# Differential phosphorylation determines the repressor and activator potencies of GLI1 proteins and their efficiency in modulating the HPV life cycle

**DOI:** 10.1371/journal.pone.0225775

**Published:** 2019-11-26

**Authors:** Alla Piirsoo, Anne Pink, Lagle Kasak, Martin Kala, Sergo Kasvandik, Mart Ustav, Marko Piirsoo

**Affiliations:** 1 Institute of Technology, University of Tartu, Tartu, Estonia; 2 Department of Chemistry and Biotechnology, Tallinn University of Technology, Tallinn, Estonia; Texas Tech University Health Sciences Center School of Medicine Lubbock, UNITED STATES

## Abstract

The Sonic Hedgehog (Shh) signalling pathway plays multiple roles during embryonic development and under pathological conditions. Although the core components of the Shh pathway are conserved, the regulation of signal transduction varies significantly among species and cell types. Protein kinases Ulk3 and Pka are involved in the Shh pathway as modulators of the activities of Gli transcription factors, which are the nuclear mediators of the signal. Here, we investigate the regulation and activities of two GLI1 isoforms, full-length GLI1 (GLI1FL) and GLI1ΔN. The latter protein lacks the first 128 amino acids including the conserved phosphorylation cluster and the binding motif for SUFU, the key regulator of GLI activity. Both GLI1 isoforms are co-expressed in all human cell lines analysed and possess similar DNA binding activity. ULK3 potentiates the transcriptional activity of both GLI1 proteins, whereas PKA inhibits the activity of GLI1ΔN, but not GLI1FL. In addition to its well-established role as a transcriptional activator, GLI1FL acts as a repressor by inhibiting transcription from the early promoters of human papillomavirus type 18 (HPV18). Additionally, compared to GLI1ΔN, GLI1FL is a more potent suppressor of replication of several HPV types. Altogether, our data show that the N-terminal part of GLI1FL is crucial for the realization of its full potential as a transcriptional regulator.

## Introduction

The Hedgehog (Hh) pathway is a conserved signal transduction system required for embryonic development and adult life in many taxa of the animal kingdom. Inappropriate regulation of the pathway leads to the progression of various developmental abnormalities and different diseases, including various forms of cancer in humans.

In mammals, the nuclear mediators of the pathway are Gli transcription factors (Gli1-3). The prevailing model of Gli protein action states that in the absence of an extracellular Hh signal, the full-length Gli2 and Gli3 (Gli2/3FL) proteins are sequestered in the cytoplasm by a protein complex containing Suppressor of fused (Sufu), the key regulator of Gli activities. Additionally, Gli2/3FL are targeted for partial degradation to produce C-terminally truncated transcriptional repressors. In response to the Hh signal, the cytoplasmic complex dissociates, Gli2FL enters the nucleus and acts as a transcriptional activator. One of the first target genes of Gli2 is *Gli1*, which encodes a potent transcriptional activator and the main amplifier of the Hh signal. Gli3FL is mainly processed to a transcriptional repressor and acts as a balancer of the signal strength (reviewed in [1]).

In humans, however, the above-described model is not fully applicable for several reasons. First, while in rodents *Gli1* gene expression is strictly ligand induced, human *GLI1* is normally expressed in many tissues and cell lines without HH signal. This would mean that either all of the expressed GLI1 protein is sequestered in the cytoplasm or some expression of HH target genes is realized in a ligand-independent manner. Indeed, it has been shown that *GLI2* and *GLI1* are transcriptional targets of the TGFβ/SMAD pathway [2][3]. Second, unlike in rodents, the human *GLI1* gene codes for at least three alternatively spliced isoforms. In addition to the *GLI1* isoform 1 (NCBI accession number NM_005269.3) encoding the GLI1FL, one isoform, named *tGLI1* (NCBI accession number NM_001167609.1), lacks exon 3 and part of exon 4 of the *GLI1* gene, but the protein retains all identified functional domains. The expression of *tGLI1* is highly specific to certain types of cancer cells [4][5]. Another isoform, termed *GLI1ΔN* (NCBI accession number NM_001160045.1), lacks the N-terminal 128 amino acids, including the SUFU binding domain and degradation signal. It has been shown that the activity of GLI1ΔN is repressed by SUFU to a lesser extent compared to GLI1FL [6]. Therefore, it is plausible to speculate that to some degree, the HH-dependent gene expression programme is active in any human cell expressing GLI1 proteins. Further description of GLI1ΔN properties and activities is needed to elucidate its role in the realization of the HH signalling cascade.

Several serine/threonine kinases are involved in the regulation of the activities of Gli proteins. For instance, the model of Gli2/3FL proteolysis establishes protein kinase A (Pka)-, glycogen synthase kinase 3-, and casein kinase 1-mediated phosphorylation as a prerequisite for the proteasomal degradation of Gli2FL and Gli3FL. The phosphorylation sites for these kinases are situated within a cluster located in the C-terminal part of the Gli2/3FL proteins after the DNA-binding zinc-finger (ZnF) domain. This cluster is less conserved in Gli1. Additionally, Pka has been suggested to phosphorylate the N-terminal Pc-g cluster of Gli2 and Gli3, which is situated in the proximity of the minimal Sufu-binding motif before the ZnF domain [7]. It has been shown that phosphomimetic mutations at specific serine or threonine residues situated within the Pc-g cluster enhance Gli2/3 transcriptional activities. In contrast, the respective non-phosphorylatable mutations lead to a decrease in Gli2/3 transcriptional activities, suggesting that this kind of phosphorylation may be required for the generation of Gli transcriptional activators. However, it has not been demonstrated convincingly that Pka is directly responsible for the phosphorylation of the Pc-g cluster. Furthermore, genetic evidence suggests that Pka has a negative impact on the activity of Gli proteins (reviewed in [8]).

The kinase suggested to be a potent positive regulator of Gli protein activities is unc-51-like kinase 3 (Ulk3). It has been shown that Ulk3 directly phosphorylates Gli proteins, stimulates Gli1/2 transcriptional activities and sustains their stability in mouse and human cells [9][10]. However, neither PKA nor ULK3 activities have been explored in the context of GLI1ΔN regulation.

In the present article, we analysed the role of PKA and ULK3 protein kinases in the regulation of the transcriptional activity of GLI1FL and the N-terminally truncated variant GLI1ΔN in SHH-responsive HUH7 cells. We show that PKA-mediated phosphorylation has differential effects on GLI1FL and GLI1ΔN. In contrast, ULK3 potentiates the activity of both proteins, further confirming its previously established role as a positive regulator of GLI proteins. Also, we explore the capability of GLI1FL and GLI1ΔN to regulate the replication of several types of human papillomaviruses (HPVs) and establish that in certain settings, GLI1FL can act as a transcriptional repressor.

## Materials and methods

### Reagents and constructs

The construct encoding the Flag-tagged PKA catalytic subunit Cα was a kind gift from Dr. Mari Sepp. The construct encoding the Flag-tagged catalytically deficient PKA catalytic subunit Cα bearing a point mutation in amino acid residue 197 (PKA(T197A)) was generated by PCR mutagenesis. Constructs encoding Flag-tagged GLI1FL, ULK3 and ULK3(K139R) are described in [9]. The HPV5, HPV11, HPV18 and HPV18E1^-^ genomes are previously described [11][12][13]. The HPV5NLuc, HPV11NLuc and HPV18NLuc genomes are described in [14]. The Flag-tagged GLI1ΔN encoding construct (a kind gift from Dr. Peter Zaphiropoulos) is described in [6]. The Myc-tagged SUFU encoding construct is described in [15]. The 8xGLI-FFluc reporter plasmid is described in [16]. The pRL-TK plasmid was purchased from Promega.

TGF-β was purchased from Peprotech. SAG (synthesized at Karolinska Institutet, Department of Biosciences and Nutrition, Novum) was dissolved in DMSO.

### Cell culture

Cell lines 293FT, A549, 1321 N1, Shh-Light2 (Shh-L2) and U2OS were purchased from ATCC. HUH7 cells were a kind gift from Dr. Pirjo Spuul. Adult pooled normal human epithelial keratinocytes (NHEKs, PromoCell) (kind gift from Dr. Ana Rebane) were grown in Defined Keratinocyte-SFM Medium (DKSM) (Gibco, Thermo Fisher Scientific). 293FT, A549, 1321 N1, and Shh-L2 cell lines were cultured in Dulbecco's Modified Eagle Medium-high glycose (DMEM, Pan Biotech) supplemented with 10% foetal calf serum (FCS, Pan Biotech) and 1% penicillin/streptomycin (PEST, Sigma-Aldrich). The growth medium of Shh-L2 cells also contained 0.4 mg/ml G418 (Sigma-Aldrich) and 0.1 mg/ml zeocine (Invitrogen). U2OS cells were propagated in Iscove's Modified Dulbecco's Medium (IMDM, Pan Biotech), 10% FCS and 1% PEST. Cells were propagated at 37°C in 5% CO_2_. HUH7 cells were transfected using Lipofectamine 2000 (Life Technologies). 293FT and Shh-L2 cells were transfected using polyethylenimine (PEI) as previously described [9]. U2OS cells were co-transfected with HPV minicircle genomes and GLI1FL or GLI1ΔN encoding constructs by electroporation (220 V and 975 μF) using a Gene Pulser XCell system (Bio-Rad Laboratories).

### Immunofluorescence (IF) microscopy

Transfected U2OS and HUH7 cells were grown on cover glasses or chamber slides (Corning) for 36 h, washed twice with washing buffer (0.5% BSA in PBS) and fixed with ice-cold methanol for 10 min at -20 ^o^C (U2OS cells) or 4% PFA for 30 min at RT (HUH7 cells). HUH7 cells were permeabilized using 0.1% Triton X-100 diluted in blocking solution (2% BSA in PBS) for 15 min at RT. The cells were washed three times and blocked overnight at 4 ^o^C. The antibodies were diluted in PBS supplemented with 1% BSA and 0.05% Triton X-100. The cells were incubated with the primary antibody (rabbit anti-Flag 1:1000, Sigma-Aldrich) overnight at 4 ^o^C, washed three times for 15 min and incubated with the secondary antibody (anti-rabbit IgG conjugated with Alexa 568 (U2OS cells), or Alexa 488 (HUH7 cells), 1:1000, Invitrogen) for 1 h at RT. Next, the cells were washed 3 times for 15 min, rinsed with Milli-Q water, and mounted using SlowFade Diamond with DAPI (Invitrogen). Images were collected using the confocal microscope LSM710 (Zeiss) with 63x APOCHROMAT oil objective lens, and analysed with ZEN2011 software.

### Luciferase assay

The Shh-L2 cells were transfected using 2 μg PEI, 250 ng of a GLI1 expression construct, 125 ng of the SUFU encoding plasmid, and 150 ng of pLacZ or respective empty vectors per well of 24-well plates. The cells were incubated for 48 h and lysed in Passive Lysis Buffer (Promega). FFluc and β-galactosidase activities were measured using the Luciferase assay kit (Biotherma) and Tropix Galacton-Light Plus (Applied Biosystems), respectively.

HUH7 cells were transfected with 1 μl Lipofectamine 2000 and 500 ng DNA per well of 48-well plates (25 ng of GLI1 encoding constructs, 250 ng of the kinase expression constructs, 100 ng of the FFluc reporter plasmid containing the 8xGLI binding site, and 50 ng of pGL4.83-hRLuc-PGKprom plasmid). The cells were incubated for 48 or 72 h. FFluc and Renilla luciferase activities were measured using the Dual Glo Luciferase Assay system (Promega).

NLuc assays were performed as described previously [14].

### Overexpression experiments

The following amounts of plasmids were used per well of a 6-well plate for transfection of HUH7 cells: 100 ng of GLI1FLpCMV-Flag-4 or GLI1ΔNpCMV-Flag-4; 500 ng of PKAwt, PKAT197A, ULK3, and ULK3(K139R) encoding constructs; and 1400 ng of pCMV-Flag-4 empty vector as a carrier. HUH7 cells were incubated for 48 h, then lysed in 100 μl of RIPA buffer (50 mM Tris pH 8.0, 150 mM NaCl, 1% NP-40, 0.5% sodium deoxycholate, 0.1% SDS) supplemented with protease inhibitor cocktail (Roche), 1 mM Na_3_VO_4_, and 10 mM NaF (both Sigma-Aldrich) and finally subjected to immunoblotting assay.

Approximately 2x10^6^ U2OS or HUH7 cells were transfected with 2 μg of the constructs encoding GLI1FL or GLI1ΔN and 500 ng of a PKA encoding construct, if indicated. Nuclear and cytoplasmic extracts were prepared using NE-PER Nuclear and Cytoplasmic Extraction Reagents (Thermo Fisher Scientific) and subjected to WB or EMSA.

### Immunoblotting assay

The following antibodies (Abs) and their dilutions were used: GLI1 rabbit (V812) (Cell Signaling Technology) 1:1000, GAPDH (Millipore) 1:10000, Flag M2 (Sigma-Aldrich) 1:5000, tubulin 1:1000 (Sigma-Aldrich), lamin A/C (clone 14) (Upstate) 1:2000, and SUFU (H300) (Santa Cruz Biotechnologies) 1:1000. HRP-conjugated secondary Abs were diluted 1:10000 (Jackson ImmunoResearch). All Abs except GLI1 V812 were diluted in PBS containing 0.1% Tween (PBST) and 2% whey. GLI1 V812 Ab was diluted in PBST containing 5% BSA. The images were obtained using the SuperSignal West Pico Chemiluminescent Substrate kit (Thermo Fischer Scientific).

### Immunoprecipitation

For IP of endogenous GLI1, A549, 1321N1 and 293FT adherent cells were lysed in IP buffer (50 mM Tris pH 8.0, 150 mM NaCl, 0.5% NP-40, 0.5% TritonX-100, 0.1% SDS, 5 mM EDTA) supplemented with protease inhibitor cocktail (Roche). Lysates were incubated on ice for 30 min and cleared by centrifugation at 14000 rpm for 25 min at 4 ^o^C. Endogenous GLI1 was immunoprecipitated using GLI1 V812 Ab (5 μl per reaction) and protein A Sepharose beads (GE Healthcare) or Dynabeads protein A magnetic beads (Invitrogen) at 4 ^o^C overnight. The immunocomplexes were washed with TBS and subjected to WB (1/5 of the sample) or MS analysis (4/5 of the sample).

293FT cells were transfected using 2 μg of the constructs encoding Flag-tagged GLI1FL, GLI1ΔN, PKA, and ULK3, incubated for 2 days and lysed in 50 mM Tris HCl, pH 7.4 supplemented with 150 mM NaCl, 1 mM EDTA, and 1% TRITON X-100 for 30 min on ice. The lysates were incubated with Anti-Flag M2 Affinity Gel (Sigma-Aldrich). Endogenous SUFU was immunoprecipitated using -SUFU Ab (C-15) conjugated to agarose (Santa Cruz Biotechnology). The obtained immunocomplexes were washed with TBS and subjected to WB and *in vitro* kinase assay.

### Total DNA isolation and SB

The following amounts of HPV genomes and GLI1 expression vectors were used for transfection of 10^6^ U2OS cells: HPV5 1500 ng, HPV11 450 ng, HPV18 900 ng, GLI1FL and GLI1ΔN or the respective empty vector 300 ng. The cells were incubated for 2, 3 or 4 days and lysed in Proteinase K buffer (20 mM Tris-HCl pH 8.0, 100 mM NaCl, 0.1 mM EDTA, 0.2% SDS). Total DNA isolation, Southern transfer and hybridization were performed as previously described [17]. The following amounts of total DNA were used for the detection of different HPV genomes: HPV5–5 μg; HPV11–2 μg; and HPV18–5 μg. Total DNA was treated with the DpnI restriction enzyme and with the following restriction endonucleases to linearize HPV genomes: SacI (HPV5), HindIII (HPV11), and BglI (HPV18). All SB assays were performed at least three times, and representative images are shown. SB signals corresponding to the replicated HPV genomes were quantified using ImageQuant software.

### RNA isolation and quantitative PCR

Approximately 10^6^ U2OS cells were used for total RNA isolation. HUH7 cells were seeded on 6-well cell culture plates, incubated for 24 h in normal growth medium and treated with 10 ng/ml TGF-β, or 10 nM SAG diluted in basic growth medium supplemented with 0.5% FBS and 1% PEST for 16 h. Total RNA was isolated using RNeasy Mini kit (Qiagen) and complementary DNA was synthesized using Superscript III reverse transcriptase (Invitrogen) and oligo(dT).

The expression levels of *GLI1* and *GLI1ΔN* were analysed in 13 human cell lines using quantitative PCR (qPCR) and a cDNA panel of 11 human cell lines (a kind gift from prof. Tõnis Timmusk) or cDNAs synthesized using total RNA isolated from U2OS and NHEK cells.

To analyse the level of HPV18E1- derived transcripts, U2OS cells were co-transfected with HPV18E1^-^ genome and GLI1FL or GLI1ΔN encoding constructs and incubated for 48 h. Total RNA was treated with Turbo DNase (Invitrogen) for 6 h and subsequently precipitated with 7.5 M LiCl. Complementary DNA was synthesized using 1.2 μg of total RNA, oligo(dT) and RevertAid First Strand cDNA Synthesis Kit (Thermo Fisher Scientific).

qPCR was performed in triplicate using LightCycler 480 SYBR Green I Master kit (Roche). The data were analysed using the ddCt method with *GAPDH* as a reference gene. The following primers were used: *GLI1FL* s CAGTTATGGGCCAGCCAGAGAG, *GLI1ΔN* s GCGCCCAGACAGAGGCCCACT, *GLI1FL/GLI1ΔN* as CATCCGACAGAGGTGAGATGGAC. Primers used for analysis of HPV18E1^-^ transcripts and *GAPDH* are described in [14].

### *In vitro* kinase assay

The *in vitro* kinase assay was performed using immunopurified Flag-tagged GLI1FL, GLI1ΔN, PKA and ULK3 proteins as previously described [9].

### Cell cycle analysis

Approximately 10^6^ U2OS cells were co-transfected with 1 μg of the HPV18 genome and 300 ng of constructs encoding GLI1FL, GLI1ΔN or an empty vector. Cell cycle profiles were analysed as previously described 3 days post-transfection [18].

### EMSA

EMSAs were performed as described previously [19]. Double stranded oligonucleotides were labelled with ^32^P using T4 polynucleotide kinase. Sequences of the sense strands of the oligonucleotides were: *GLIRE* AGCTACCTGGGTGGTCTCTTCGA and *POURE* GAGAGGAATTTGCATTTCCACCGACCTTCC.

## Results

### The GLI1 locus codes for proteins with- and without the N-terminal Pc-g cluster

**Fig 1:**
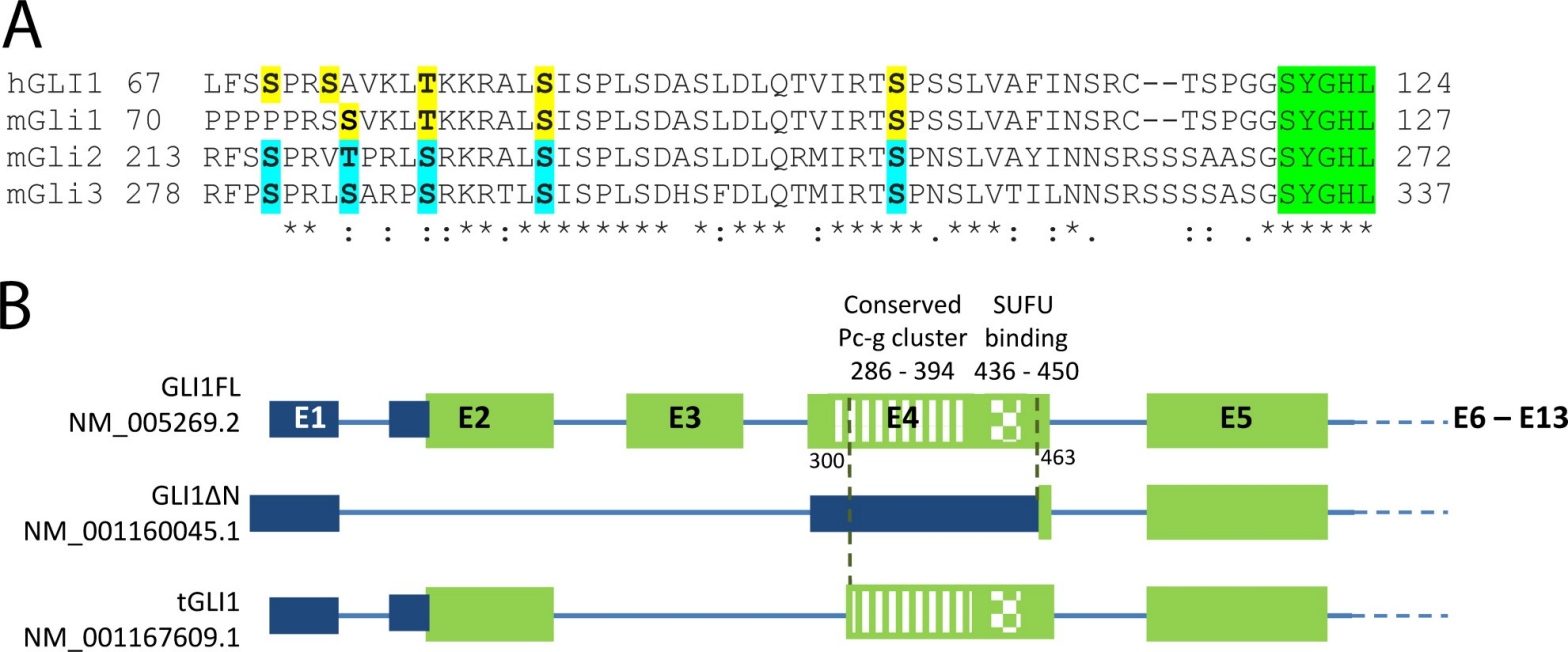
GLI1FL, but not GLI1ΔN, possesses a conserved Pc-g cluster and SUFU binding motif encoded within exon 4. (A) Sequence alignment of the N-terminal parts of human GLI1 (hGLI1) and mouse mGli1, mGli2 and mGli3 was performed using Clustal Omega software. The serine or threonine residues of mGli2 and mGli3 located within the Pc-g cluster and suggested as phospho-acceptor sites for PKA-mediated phosphorylation are indicated by blue. The respective residues in hGLI1 and mGli1 are shown in yellow. The conserved residues crucial for the interaction with Sufu are indicated in green. (B) A simplified schematic presentation of the GLI1 gene structure. The GLI1 gene consists of 13 exons (E). The protein encoding part is indicated in green, and the non-coding region is shown in blue. The sequences encoding the Pc-g cluster and the residues required for interaction with SUFU are indicated in exon 4 of GLI1FL (nucleotides 284–401 and 439–451, respectively). Due to the alternatively spliced exons, the ORF of GLI1ΔN starts from the late ATG corresponding to nucleotides 463–465 of GLI1FL. GLI1ΔN lacks the first 128 amino acids containing the Pc-g cluster and the SUFU-binding motif.

Phosphorylation of Gli proteins is a prerequisite for a wide range of regulatory processes varying from the full or partial degradation and generation of the C-terminally truncated transcriptional repressors to stimulation of the transcriptional activity and nuclear translocation of the full-length proteins. The significance of the Pc-g phosphorylation cluster for the transcriptional activity of mouse Gli2/3 proteins has been described previously [7]. Here, we were interested in exploring whether the Pc-g cluster is also conserved in human GLI1 proteins. Sequence alignment of the N-terminal parts of human GLI1 and mouse Gli1, Gli2 and Gli3 proteins revealed that the Gli1 proteins contained the Pc-g cluster. Additionally, the serine residues suggested as phosphoacceptor sites for the PKA-mediated phosphorylation were well conserved (Fig 1A).

Since human *GLI1* has at least three alternatively spliced isoforms, *GLI1FL*, *tGLI1* and *GLI1ΔN*, we explored whether the resulting proteins contained all important regulatory domains including the Pc-g cluster. The minimal SUFU-binding motif and almost the entire Pc-g phosphorylation cluster were present in tGLI1. In contrast, these regulatory elements were absent in GLI1ΔN, the open reading frame (ORF) of which started immediately before the sequence encoding the ZnF domain (Fig 1B). Thus, our bioinformatics analysis suggests that the N-terminally truncated GLI1ΔN, lacking amino acids 1–128, may be regulated via a completely different mechanism compared to GLI1FL/tGLI1. The existence of GLI1FL and tGLI1 proteins has been shown experimentally, but there have been difficulties in proving the existence of a GLI1ΔN protein. However, the GLI1ΔN transcript contains a reasonably good Kozak consensus sequence around its initiator ATG, indicating that the difficulties in proving the existence of the protein might be due to the lack of suitable antibodies and/or low expression levels.

### GLI1FL and GLI1ΔN proteins are expressed in a variety of human cell lines

**Fig 2:**
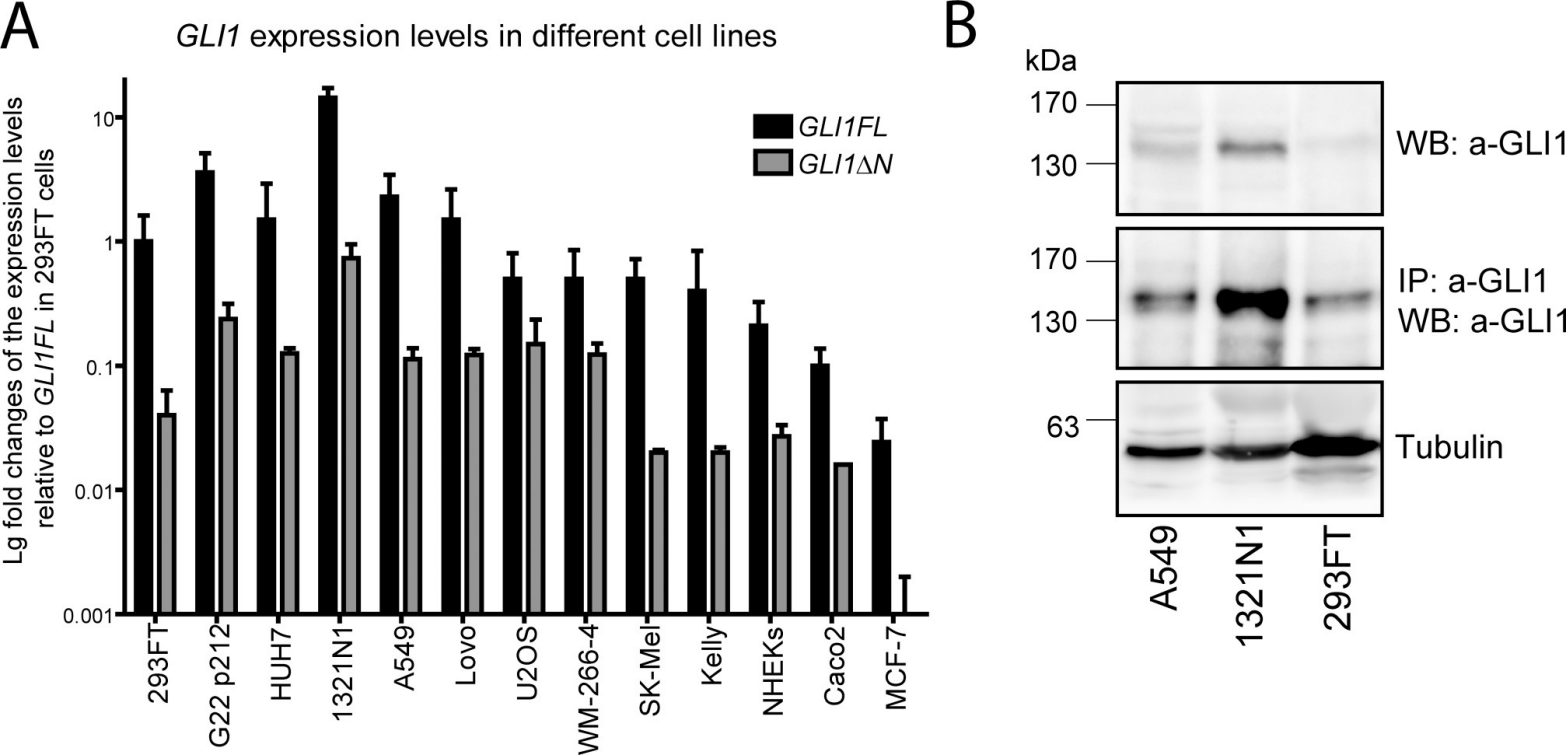
GLI1FL and GLI1ΔN are co-expressed in a wide variety of cell lines. (A) *GLI1FL* and *GLI1ΔN* mRNA expression levels in different cell lines were measured using qPCR, normalized to *GAPDH* expression and set as 1 in the 293FT cell line. Data are presented as the average mean +/- SD. (B) The endogenous GLI1 proteins were analysed using immunoblotting in the cell lines expressing different levels of GLI1FL/GLI1ΔN mRNAs. The levels of GLI1 proteins were examined either in whole-cell extracts (upper panel) or in the immuno-complexes precipitated using a-GLI1 (V812) antibody and beads conjugated with protein A (middle panel). Tubulin was used as a loading control.

The expression of *GLI1ΔN* has been analysed previously in selected rhabdomyosarcoma, prostate, pancreas and lung cancer cell lines [6]. We expanded this choice of cell lines and analysed the mRNA expression of *GLI1FL* and *GLI1ΔN* transcripts in thirteen human cell lines using qPCR (Fig 2A). The tumour types covered were glioblastoma (G22 p212), hepatocellular carcinoma (HUH-7), astrocytoma (1321N1), lung carcinoma (A549), colon carcinoma (Lovo, Caco), osteosarcoma (U2OS), melanoma (SK-MEL, WM-266), neuroblastoma (Kelly), and breast cancer (MCF-7). Also, we analysed the levels of *GLI1FL* and *GLI1ΔN* mRNAs in normal human epithelial keratinocytes (NHEKs) and a clone of human embryonal kidney 293FT cells. *GLI1FL* and *GLI1ΔN* transcripts were detected in all analysed cell lines. The normalized level of *GLI1FL* mRNA expression in the control cell line 293FT was set as 1. The highest *GLI1FL* mRNA expression level was detected in the 1321 N1 cell line (approximately 14 times over control). The A549 cell line demonstrated a moderate *GLI1FL* mRNA expression level (approximately 2.3 times over the control). The lowest level of *GLI1FL* expression was found in Caco-2 and MCF-7 cell lines (approximately, 12 and 41 times under control). Generally, the levels of *GLI1FL* and *GLI1ΔN* mRNAs showed a good correlation (R2 = 0.953) (S1 Fig), though the *GLI1FL* mRNA expression level exceeded that of *GLI1ΔN* approximately 15 times in all analysed cell lines. Nevertheless, the *GLI1ΔN* expression level in six cell lines (G22 p212, HUH7, 1321 N1, A549, Lovo, and WM-266-4) was higher than or comparable to the *GLI1FL* mRNA expression level in the Caco-2 and MCF-7 cell lines.

Next, we analysed the levels of GLI1 proteins in the cells that demonstrated the highest levels of *GLI1FL* and *GLI1ΔN* mRNA expression (1321 N1, A549 and 293FT) using immunoblotting and an antibody against GLI1 protein (Fig 2B). Since GLI1 was barely detectable even in the whole-cell extracts of 1321N1 cells, we immunoprecipitated the GLI1 proteins and analysed the immunocomplexes using Western blot (WB). In both cases, the GLI1 protein(s)-specific signal was detected between 130 and 170 kDa. Nevertheless, it was difficult to distinguish whether GLI1 migrated as a single or a double band. Therefore, the GLI1 immunocomplexes purified from the 1321 N1 cell line were subjected to trypsinolysis and subsequent mass spectrometry (MS) analysis to examine the presence of the GLI1ΔN N-terminal peptide MSPSLGFPAQMNHQK (S2 Fig). Our analysis revealed that the peptide was present in the analysed immunocomplexes, indicating that the GLI1ΔN protein is expressed in 1321 N1 cells.

### PKA inhibits GLI1ΔN transcriptional activity in a kinase activity-dependent manner

**Fig 3:**
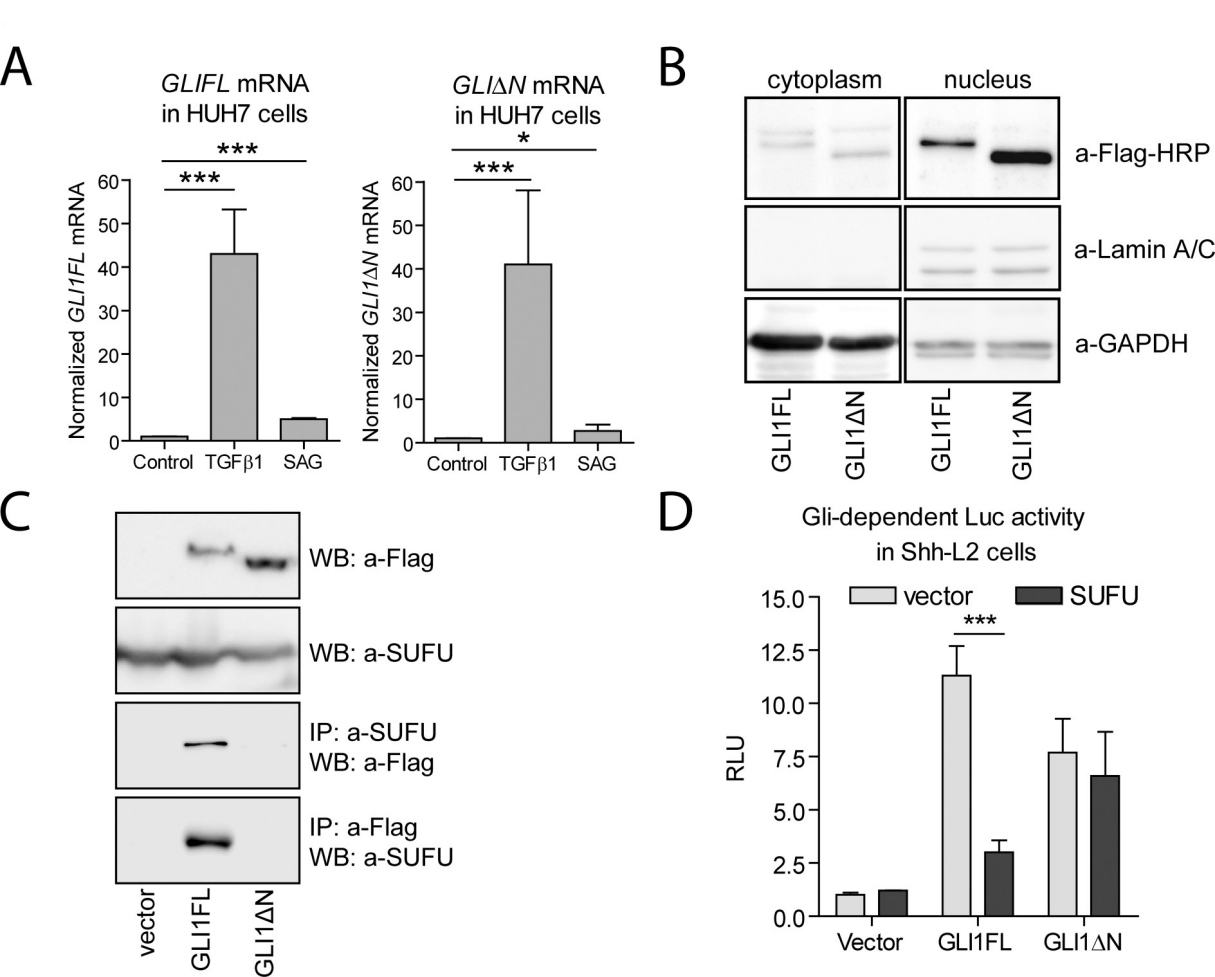
In contrast to GLI1FL, the GLI1ΔN protein does not interact with SUFU. (A) HUH7 cells were treated with 10 ng/ml TGFβ1 or 10 nM SAG for 24 h. The levels of GLI1FL and GLI1ΔN mRNAs were measured using qPCR, normalized to GAPDH mRNA expression level and set as 1 in the control cells treated with DMSO. (B) Flag-tagged GLI1FL and GLI1ΔN proteins were overexpressed in HUH7 cells. Levels of cytoplasmic and nuclear GLI1FL, GLI1ΔN, GAPDH and LAMIN A/C were detected using WB. (C) Endogenous SUFU and overexpressed Flag-tagged GLI1FL and GLI1ΔN proteins were immunoprecipitated and subjected to WB analysis using a-Flag-M2 and a-SUFU (H300) antibodies. (D) GLI1FL and GLI1ΔN proteins were overexpressed with SUFU or an empty vector in Shh-L2 cells. The cells were incubated for 72 h and subjected to a luciferase assay. Normalized FFLuc activity in the cells transfected with the empty vector was set as 1. All panels (if applicable): data are presented as the average mean +/- SD. The two-tailed p value was calculated using Excel software (*—p<0.05, ***—p<0.001).

Assuming that GLI1FL and GLI1ΔN may be regulated differently due to their unique N-termini, we were interested in examining the GLI1FL and GLI1ΔN activities in a HH-responsive human cell line. Generally, the canonical Hh/Gli pathway is studied in mouse cells, such as C3H10T1/2, NIH3T3 or MEFs, where Gli1 transcription is a readout of the Hh pathway activity. In human cells, expression of *GLI1* can be induced by TGFβ in a GLI2-dependent manner, whereas treatment with HH is often inefficient. Among the tested cell lines, only HUH7 cells responded to the SAG (agonist of the HH co-receptor SMOOTHENED) and TGFβ treatment, demonstrating upregulation of *GLI1FL* and *GLI1ΔN* mRNA expression levels in response to each inducer (Fig 3A).

GLI1 has been found in both the nucleus and the cytoplasm of different human cell lines. Shuttling of the GLI1 protein between cytoplasmic and nuclear compartments depends on several factors including interaction with SUFU and PKA-mediated phosphorylation [15][20]. We examined the levels of over-expressed GLI1FL and GLI1ΔN proteins in the nuclear and cytoplasmic fractions of the HUH7 cells using WB (Fig 3B). Both proteins were detected in cytoplasmic and nuclear extracts at between 130 and 170 kDa. These results were confirmed using IF analysis, which showed that the overexpressed GLI1FL and GLI1ΔN proteins were localized in the nuclear and cytoplasmic compartments (S3 Fig). The level of GLI1ΔN in the nuclear fraction exceeded that of GLI1FL, which might have been caused by the inability of GLI1ΔN to interact with SUFU that sequesters GLI proteins in the cytoplasm. Consistent with the previously reported results [6], we also observed a severely reduced capacity of GLI1ΔN to interact with SUFU in the co-immunoprecipitation and luciferase assays (Fig 3C and 3D).

**Fig 4:**
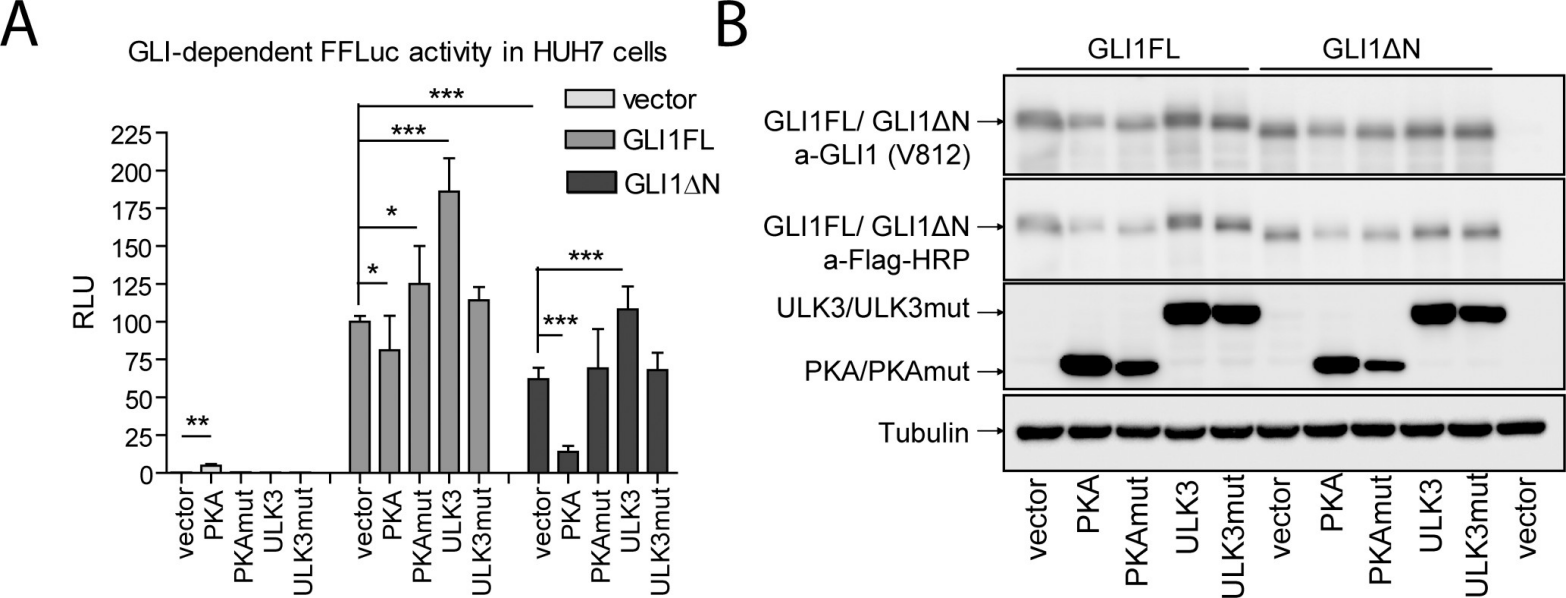
Transcriptional activity of GLI1FL and GLI1ΔN is regulated by the kinases PKA and ULK3. (A) GLI1FL and GLI1ΔN proteins were overexpressed with the catalytically active PKA or ULK3, kinase activity-deficient mutant PKA or ULK3 or an empty vector in HUH7 cells in the presence of 8xGli-FFLuc and pRL-TK reporter plasmids. GLI-dependent FFLuc activity was normalized to the Renilla Luc activity and set as 1 in the samples transfected with the empty vector. The data are presented as the mean +/- SD. Statistical analysis was performed and the two-tailed p values were calculated using Excel software (*—p < 0.05, ***—p < 0.001). (B) HUH7 cells were transfected with constructs encoding Flag-tagged GLI1FL or GLI1ΔN along with Flag-tagged catalytically competent PKA or ULK3, kinase activity-deficient mutant PKA or ULK3 or the respective empty vector. Whole cell extracts were subjected to WB analysis using a-Flag-HRP, a-GLI1 and a-tubulin antibodies.

To analyse the impact of PKA and ULK3 kinases on the transcriptional activity of GLI1FL and GLI1ΔN, HUH7 cells were co-transfected with GLI1FL or GLI1ΔN-encoding constructs together with an 8xGli-dependent luciferase reporter and catalytically active PKA or ULK3 or their kinase activity-deficient mutants and subjected to luciferase assay (Fig 4A). Our analysis showed that the potency of GLI1ΔN to act as a transcriptional activator was approximately 2 times lower than that of GLI1FL, which is consistent with the previously reported data from Shimokawa and colleagues obtained in Shh-responsive mouse cells NIH3T3 [6]. The PKA catalytic subunit alone slightly stimulated the activity of the endogenous GLI proteins. However, the overexpressed GLI1FL and GLI1ΔN demonstrated a reduced capacity to activate transcription in the presence of PKA. Interestingly, the intensity of the PKA-mediated inhibition was different between the two proteins. The transcriptional activity of GLI1ΔN was reduced more than 5 times, but the activity of GLI1FL was inhibited only 25%. In contrast, ULK3 induced transcriptional activity of both GLI1FL and GLI1ΔN by approximately 75%. Both kinases, PKA and ULK3, regulated GLI1FL and GLI1ΔN transcriptional activities in a kinase activity-dependent manner, while their mutants did not alter GLI1FL or GLI1ΔN-dependent luciferase activity in HUH7 cells (Fig 4A).

One possible reason that could explain the effects of PKA and ULK3 on the transcriptional activity of GLI1 proteins is that the both protein kinases can alter the expression level and/or stability of the proteins. To examine the levels of the overexpressed GLI1FL and GLI1ΔN proteins in the presence of catalytically active PKA or ULK3 or their kinase-deficient mutants, whole-cell extracts of the transfected HUH7 cells were subjected to immunoblotting assay (Fig 4B). Our analysis showed that the expression levels of the GLI1FL and GLI1ΔN transcription factors were affected by the co-expressed proteins. The levels of both GLI1FL and GLI1ΔN proteins were similar in the presence of PKA or its catalytically deficient mutant. However, a slight upward shift in GLI1FL and GLI1ΔN migration in the presence of the PKA catalytic subunit suggested that both transcription factors might be phosphorylated by PKA in HUH7 cells. A similar shift was also observed in the presence of catalytically active ULK3. These data indicate that the strong PKA-mediated inhibition of GLI1ΔN-induced transcription, observed in the luciferase assay, was not a result of the specific degradation or more extensive downregulation of GLI1ΔN expression compared to the GLI1FL protein.

**Fig 5:**
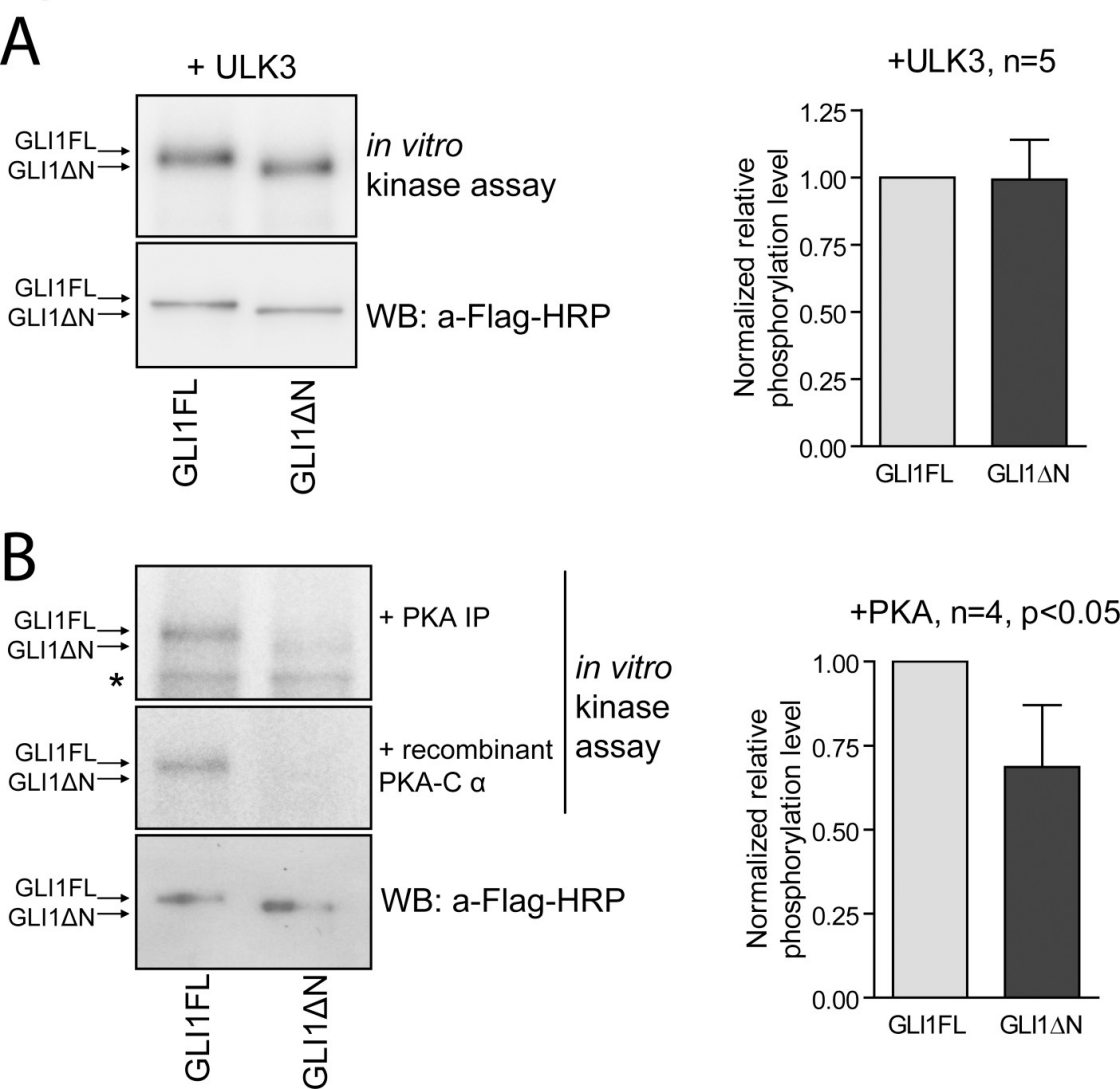
ULK3 and PKA phosphorylate GLI1FL and GLI1ΔN *in vitro*. (A) Flag-tagged ULK3, GLI1FL and GLI1ΔN were overexpressed, immunopurified and subjected to the *in vitro* kinase assay. The levels of GLI1FL and GLI1ΔN in the kinase reactions were detected using WB (left panel). The ULK3 mediated phosphorylation signals were quantified and shown relative to GLI1FL (right panel). (B) Flag-tagged PKA, GLI1FL and GLI1ΔN were overexpressed, immunopurified and subjected to the *in vitro* kinase assay (left panel, upper section). Immunopurified GLI1FL and GLI1ΔN were subjected to the *in vitro* kinase assay using bacterially purified PKA catalytic subunit (left panel, middle section). The levels of GLI1FL and GLI1ΔN in the kinase reactions were detected using WB (left panel, lower section). The PKA mediated phosphorylation signals were quantified and shown relative to GLI1FL (right panel). The two-tailed p value was calculated using Excel software; n–number of experiments; *—non-specific band.

The fact that PKA-mediated regulation of GLI1FL and GLI1ΔN activities diverged prompted us to analyse, whether PKA phosphorylates the respective GLI1 proteins differentially. We also included ULK3 kinase in the analysis to study whether the unique N-terminal part of GLI1FL is phosphorylated by this protein kinase. We overexpressed and immunopurified the GLI1 proteins, PKA, and ULK3 in 293FT cells and subjected them to an *in vitro* kinase assay. Both kinases were able to phosphorylate GLI1FL and GLI1ΔN (Fig 5). Quantitation of the intensity of phosphorylation signals showed that ULK3 phosphorylated both GLI proteins similarly (Fig 5A). However, compared to GLI1ΔN, GLI1FL was a better substrate for PKA. This difference was especially evident, when recombinant PKA catalytic subunit was used in the *in vitro* kinase assay (Fig 5B). These data suggest that PKA phosphorylation sites reside not only in the C-terminal part of GLI1FL but also in its N-terminus.

Taken together, these data indicate that PKA-mediated phosphorylation has a strong negative effect on the transcriptional activity of GLI1ΔN in HUH7 cells, whereas phosphorylation of the N-terminus of GLI1FL counterbalances this inhibitory effect. In addition, our data indicate that ULK3-mediated phosphorylation occurs at residues located outside the first 128 amino acids of the GLI1FL protein.

### GLI1FL, but not GLI1ΔN, acts as a repressor of HPV transcription

There are only a few known target genes that could be used to assess the potency of GLI1 transcriptional activators. Moreover, many of them are strictly cell type specific. Therefore, GLI-dependent reporter constructs have been routinely used to examine the transcriptional activity of GLI proteins. Often these reporters harbour multiple ideal GLI binding sites in front of a heterologous promoter, a situation which very rarely, if ever, occurs in real life.

We were interested in studying the potency of GLI1FL and GLI1ΔN as transcription factors in a more physiological setting. We observed that GLI1 mRNAs were expressed in NHEKs and U2OS cells (Fig 2A). NHEKs are native host cells for HPV infections, whereas U2OS cells are one of the few established cell lines that support the replication of several HPV genomes [17]. We decided to assess the impact of GLI1FL and GLI1ΔN on the transcription of the HPV18E1- genome in U2OS cells. This HPV genome represents a high-risk type that causes cervical cancer, and *GLI1* is expressed in several cervical carcinoma cell lines [21]. The chosen HPV18E1^-^ genome bears a point mutation in the E1 ORF and therefore lacks expression of E1 helicase, one of the viral proteins absolutely required for HPV replication [17]. Since the HPV18E1- genome is unable to replicate, direct effects on viral transcription can be examined. The consensus GLI binding site sequence GACCACCCA, first determined by Kinzler and Vogelstein [22], is missing in the HPV18 genome. However, subsequent studies have shown that non-consensus GLI binding sites, with relatively low affinity to the protein can still function as strong regulatory elements of GLI dependent transcription [23][24][25]. Such candidate sites can be also found in the non-coding, regulatory regions of several HPV types (Table 1).

**Table 1:** Putative non-consensus GLI binding sites in the noncoding regions of HPV5, HPV11 and HPV18 genomes.

HPV type	position in the genome	strand	sequence
HPV5	7479–7488	-	GTACCACTT
HPV11	7853–7862	+	ACCCACAAA
HPV11	7903–7912	+	ACCCACACC
HPV18	7291–7300	-	GAACCACAA
HPV18	7308–7317	-	CAACCACAT
HPV18	7347–7356	-	ATACCACAA
HPV18	93–102	+	CACCACAAT
GLI consensus			GACCACCCA

Both strands of the noncoding regions of the respective viral genomes were inspected for the almost invariant sequence CCAC in the GLI binding site. Nucleotides surrounding this sequence were further evaluated so that they will not inactivate the activity of the binding site according to published data [23].

**Fig 6:**
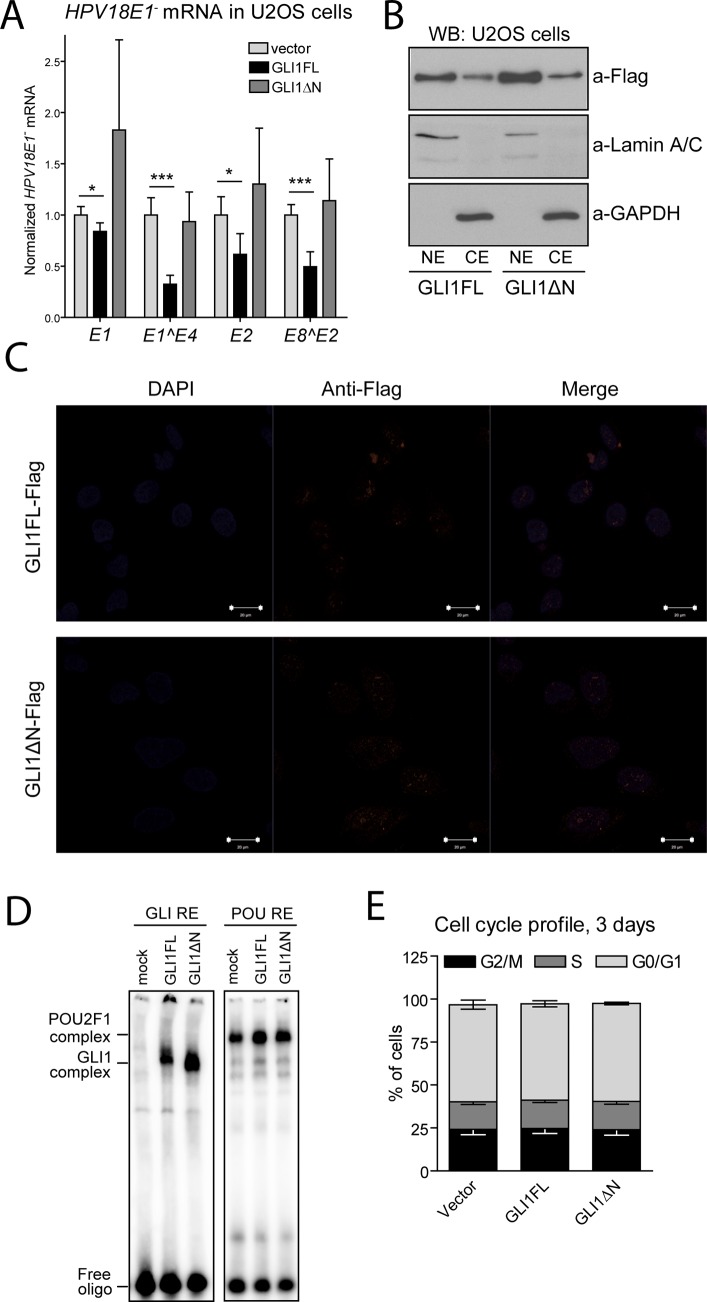
GLI1FL represses transcription from the HPV18E1- genome. (A) U2OS cells were transfected with the HPV18E1^-^ genome in combination with an empty vector or GLI1FL or GLI1ΔN construct. The levels of viral transcripts were analysed by qPCR 48 h post-transfection and normalized to *GAPDH* expression level. The levels of the HPV18E1^-^ transcripts were set as 1 in the cells co-transfected with the empty vector. The two-tailed p values were calculated using Excel software; *—p < 0.05, ***—p < 0.001, n = 3. (B) Flag-tagged GLI1FL and GLI1ΔN proteins were over-expressed in U2OS cells. The levels of GLI1FL and GLI1ΔN proteins, Lamin A/C and GAPDH were analysed in nuclear and cytoplasmic extracts (NE and CE, respectively) of U2OS cells 48 h post-transfection. (C) Flag-tagged GLI1FL and GLI1ΔN proteins were over-expressed in U2OS cells, and the subcellular localization of the respective proteins was analysed using IF microscopy 36 h after transfection. Majority of the GLI1 signal (red) was co-localized into the nucleus with the DNA stain DAPI (blue). (D) Equal amounts of nuclear extracts from the transfected U2OS cells were subjected to EMSA using GLI and POU response elements. (E) U2OS cells were co-transfected with the HPV18E1^-^ genome and GLI1FL or GLI1ΔN plasmids and incubated for 2 days. Cell cycle profile was analysed using propidium iodide by flow cytometry.

Expression vectors coding for GLI1FL and GLI1ΔN were co-transfected with the HPV18E1^-^ genome into U2OS cells, and the levels of viral transcripts were analysed by qPCR. HPV transcripts are polycistronic, and at least four viral promoters are simultaneously active in U2OS cells [26]. It is believed, however, that all these promoters are regulated similarly, with HPV-encoded transcription factor E2 being the main regulator (reviewed in [27]). We analysed the expression of two non-spliced viral mRNAs (*E1* and *E2*) and two spliced mRNAs (*E1^E4* and *E8^E2*). As shown in Fig 6A, GLI1FL repressed all HPV18 transcripts analysed, whereas GLI1ΔN failed to regulate any of them significantly.

This was a surprising result for two reasons. First, it is generally believed that GLI1 is an activator of transcription, rather than a repressor. Second, while GLI1ΔN protein is a weaker transcriptional activator compared to GLI1FL, it still had noticeable activity in the assay described earlier in this study.

Three possibilities come to mind to explain the lack of activity of GLI1ΔN in repressing HPV18 transcription. First, it is possible that for some reason, the majority of the overexpressed GLI1ΔN protein is retained in the cytoplasm in U2OS cells. It has been previously shown that overexpressed GLI1FL protein is solely nuclear, whereas GLI1ΔN is both cytoplasmic and nuclear [6]. We analysed the subcellular localization of the overexpressed GLI1FL and GLI1ΔN proteins in nuclear and cytoplasmic extracts from U2OS cells using WB and IF. As shown in Fig 6B and 6C, both proteins were present predominantly in the nuclear compartments. Similar to our results obtained using HUH7 cells, we observed that the level of nuclear GLI1ΔN exceeded that of GLI1FL in U2OS cells.

The second possibility explaining not only the inability of GLI1ΔN to repress HPV18 transcription but also its weaker potency as a transcriptional activator is an altered DNA binding activity. Although the truncated GLI1ΔN protein retains its full DNA-binding domain, the missing N-terminus may affect the DNA-binding activity. An electrophoretic mobility shift assay (EMSA) was performed to examine this hypothesis. Nuclear extracts from the transfected U2OS cells were subjected to EMSA using double-stranded oligonucleotides containing the GLI response element (RE). The amount of nuclear extracts was normalised to the level of the GLI1 protein as measured in WB analysis. As a quality control of nuclear extracts, we used the POU RE, which is shifted by the OCT1 protein in U2OS cells. Both GLI1 proteins were able to bind the GLI RE, with GLI1ΔN protein being a slightly better binder (Fig 6D).

Third, it is conceivable to speculate that since GLI1FL is a more potent transcription factor than GLI1ΔN, overexpression of it might alter the cell cycle in a way that it suppresses transcription from the HPV18E1^-^ genome. Thus, we analysed the cell cycle profiles of U2OS cells transfected with the HPV18E1^-^ genome and expression constructs coding for either GLI1FL or GLI1ΔN. Neither of the overexpressed GLI1 proteins altered the cell cycle compared to mock transfected U2OS cells (Fig 6E).

Taken together these results establish GLI1FL as a protein capable of mediating transcriptional repression. Furthermore, we can conclude that the sequences necessary for transcriptional repression lie in the N-terminus of the protein and are absent in GLI1ΔN.

### GLI1 proteins suppress replication of different HPV genomes

Next, we examined if the negative effect of GLI1FL on HPV18 transcription also manifests itself in the suppression of replication of the viral genome in U2OS cells. The logic behind this assumption lies in the fact that replication of the HPV genomes is dependent on two viral proteins, the helicase E1 and the transcription factor E2 [28]. If GLI1FL suppresses the expression of these proteins, replication of the viral genome could be less efficient, assuming the lower E1 and/or E2 levels become rate limiting in viral genome replication. To study if the GLI1FL mediated suppression of HPV replication is specific to HPV18, or is a wider phenomenon, we also included HPV types 5 and 11 in the analysis. HPV11 and -18 belong to the same alpha genus of PVs infecting mucosal epithelium, but HPV11 is a low-risk type unrelated to cervical cancer. HPV5, belonging to the genus beta, infects skin.

**Fig 7:**
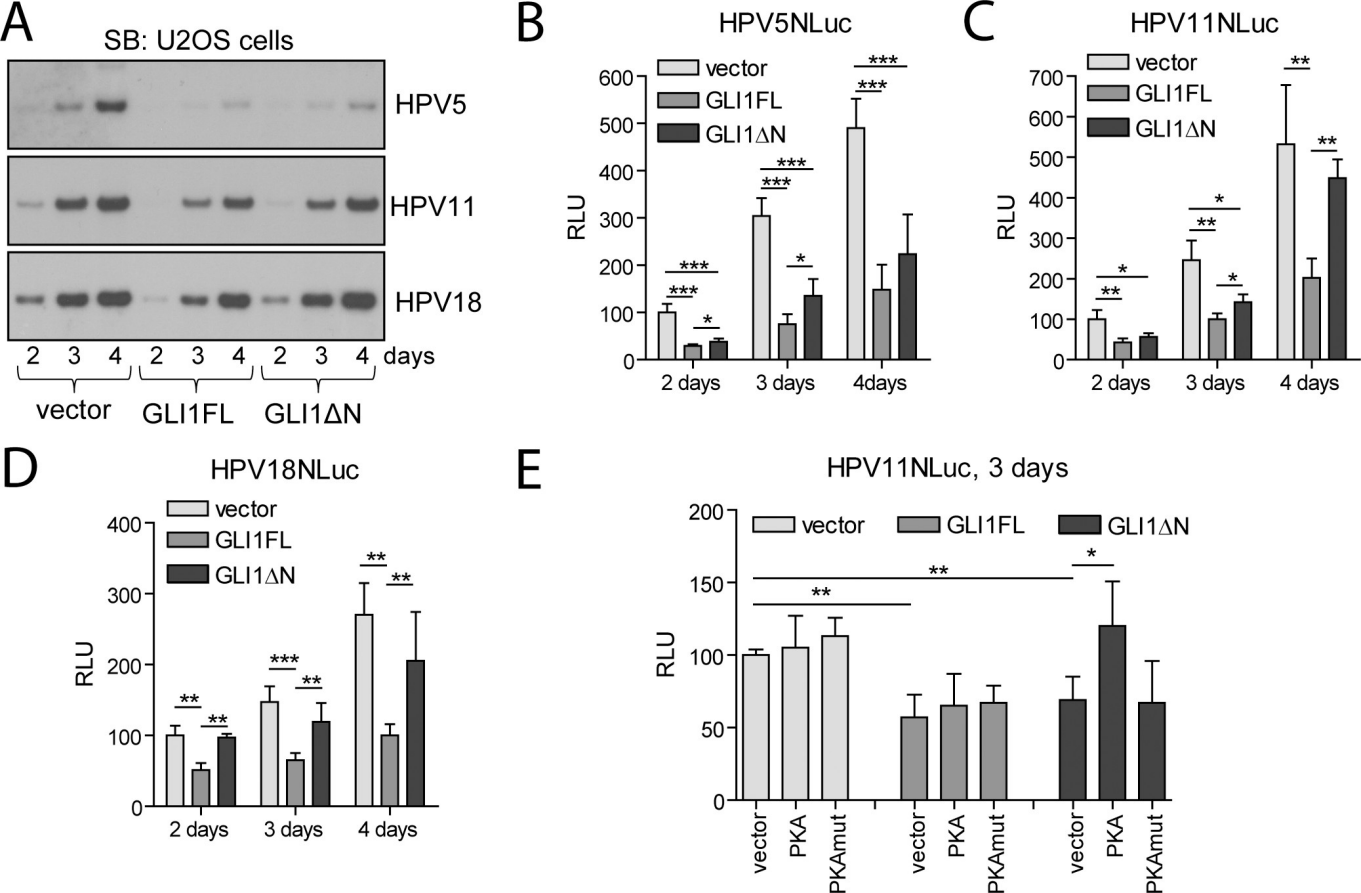
GLI1 isoforms inhibit the replication of the HPV5, HPV11 and HPV18 genomes. (A) U2OS cells were transfected with the HPV5, HPV11 and HPV18 genomes together with empty vector or plasmids encoding GLI1FL or GLI1ΔN. Total DNA was isolated 2, 3 and 4 days post-transfection, treated with DpnI and other restriction enzymes to linearize the HPV genomes, and analysed using SB. (B-D) U2OS cells were transfected with the respective HPVNLuc genomes and the construct encoding GLI1FL or GLI1ΔN (or an empty vector). NLuc activity was measured 2, 3 and 4 days post-transfection, normalized to alkaline phosphatase activity and set as 100% in the samples incubated for 2 days after transfection. Normalized NLuc data are presented as the mean +/- SD of at least 3 replicates measured in 3 independent experiments. (E) U2OS cells were transfected with the HPV11NLuc genome, the construct encoding catalytically active or deficient PKA, and GLI1FL or GLI1ΔN. NLuc activity was measured 36 h post-transfection, normalized to alkaline phosphatase activity and set as 100% in the cells transfected with the HPV11NLuc genome and the respective empty vector. NLuc data are shown as the mean +/- SD (n = 3, *—p < 0.05, **—p < 0.01, ***—p < 0.001).

The respective replication competent viral genomes were co-transfected into U2OS cells together with GLI1FL or GLI1ΔN expression vectors, and viral genome replication was analysed using Southern blotting (SB) 2, 3 and 4 days after transfection (Fig 7A). GLI1FL was able to suppress replication of all three HPV types tested, although to different degrees. It was the most potent inhibitor of HPV5 replication, and the weakest inhibition was observed in the case of HPV18. Surprisingly, GLI1ΔN was also able to inhibit HPV replication, although to a lesser extent. Quantitation of the replication signals revealed that two days after transfection GLI1FL inhibited the replication of HPV5 80%, HPV11 and HPV18 by 65%, whereas GLI1ΔN inhibited HPV5 by 55%, HPV11 by 38% and HPV18 by 32% (S4 Fig). Strong inhibition of HPV5 replication by both GLI1FL and GLI1ΔN remained throughout the whole period of analysis, whereas inhibition of HPV11 and HPV18 replication by GLI proteins became weaker over time. Similar results were obtained using the respective HPV genomes containing the Nano luciferase (NLuc) encoding sequence, whose activity represents the genome copy number [14]. The HPV5NLuc, HPV11NLuc or HPV18NLuc genome was co-transfected with a GLI1FL- or GLI1ΔN-encoding construct in U2OS cells. NLuc activity was measured 2, 3 and 4 days post-transfection and normalized to alkaline phosphatase activity (Fig 7B–7D for HPV5NLuc, HPV11NLuc and HPV18NLuc genomes, respectively). Compared to GLI1ΔN, GLI1FL inhibited replication of the HPVNLuc genomes more effectively.

To test the impact of PKA on the GLI1FL- or GLI1ΔN-mediated suppression of HPV replication, we co-transfected the respective constructs together with the HPV11NLuc genome in U2OS cells and measured NLuc activity 36 h post-transfection. HPV11NLuc was chosen as a model genome that was moderately inhibited by GLI1 proteins. Similar to the results obtained in HUH7 cells, catalytically active PKA significantly inhibited the activity of only GLI1ΔN and had no effect on GLI1FL activity. However, in this case, the PKA-mediated inhibition of GLI1ΔN activity was expressed as a reduction in GLI1ΔN-dependent suppression of HPV11NLuc replication.

## Discussion

### Differential phosphorylation determines the potencies of GLI1 isoforms to activate transcription

In the present article, we investigated the activities of two GLI1 isoforms, GLI1FL and GLI1ΔN, that are co-expressed in various human cell lines. Compared to GLI1FL, GLI1ΔN lacks the first 128 amino acids, including the SUFU binding domain and the conserved Pc-g phosphorylation cluster. All other identified functional domains, such as the ZnF, trans-activation domain, nuclear localization signal, and export signal, are present in both GLI1 proteins [8]. Therefore, it can be assumed that both proteins act as transcriptional activators with similar potency. However, the work of Shimokawa and colleagues has shown that GLI1ΔN is a much less potent transcriptional activator than GLI1FL [6]. The authors explain this phenomenon by the fact that while overexpressed GLI1FL is predominantly nuclear, considerable amounts of GLI1ΔN are retained in the cytoplasm in NIH3T3 cells. Our data do not support these conclusions. While the results asserting weaker transcriptional activity of GLI1ΔN were confirmed, we observed levels of nuclear GLI1ΔN over those of GLI1FL in both HUH7 and U2OS cells. The usage of different cell lines might explain this discrepancy, but our results also indicate that the subcellular localization is not the reason for the reduced activity of GLI1ΔN. The higher levels of nuclear GLI1ΔN might be explained by the lack of the SUFU-binding motif and failure to sequester GLI1ΔN in the cytoplasm via interaction with SUFU. Indeed, our data confirm that GLI1ΔN is unable to interact with SUFU.

Given that the subcellular localization of GLI1 proteins does not explain the reduced transcriptional activity of GLI1ΔN, we examined if phosphorylation might account for the difference. We investigated the impact of two protein kinases, ULK3 and PKA, on the GLI1FL and GLI1ΔN proteins with an emphasis on the regulation of the N-terminal Pc-g cluster of phosphorylation. Our previous studies have shown that Ulk3 is one of the few known protein kinases able to activate Gli proteins via direct phosphorylation, although the phosphoacceptor residues have remained unknown [9][10]. We also included PKA in the analysis, since it has been shown previously that PKA is the main negative regulator of Gli proteins that can phosphorylate the N-terminal Pc-g cluster of Gli3 *in vitro* [20] [29] [7]. We were interested in whether ULK3 and PKA differentially phosphorylate GLI1FL and GLI1ΔN and whether the difference in phosphorylation might contribute to the reduced transcriptional activity of GLI1ΔN.

Our results showed that ULK3 had a similar positive effect on the transcriptional activity of both GLI1 proteins analysed. Additionally, the ULK3-mediated phosphorylation efficiencies of GLI1FL and GLI1ΔN were similar in the *in vitro* kinase assay. Therefore, we can conclude that the N-terminal part of GLI1FL is dispensable for the ULK3-mediated positive effect on GLI1 activity. We can also speculate that given the equal ULK3-governed phosphorylation intensity in both GLI1 proteins, there are no ULK3 phosphorylation sites present within the first 128 amino acids of GLI1FL.

In contrast to ULK3, PKA had a clear differential effect on the activities of GLI1FL and GLI1ΔN in HUH7 and U2OS cells. PKA had a very minor negative effect on the activities of GLI1FL, whereas it almost blocked the function of GLI1ΔN either as a transcriptional activator of the Gli-dependent FFluc reporter in HUH7 cells or as a repressor of HPV11NLuc replication in U2OS cells. We also showed that PKA phosphorylated GLI1FL more profoundly than GLI1ΔN. This differential effect can be explained by the reasoning that PKA phosphorylates at least two regions in GLI1FL. One of them is also present in GLI1ΔN, which is responsible for the repression of the activator function of the protein. If the sequence around the phosphorylation site is similar to that of Gli2, then this region needs to be dephosphorylated to obtain fully active GLI1. Phosphorylation of another region counterbalances the negative effect of the first phosphorylation and is also needed for full activity of the protein. The presence of this region in GLI1FL explains the augmented transcriptional activity of GLI1FL over GLI1ΔN. We propose that the N-terminal PKA phosphorylation sites within GLI1FL lie in the Pc-g cluster. A similar model has been proposed to explain the generation of Gli2 activator and repressor forms [7].

### GLI1 isoforms and HPV life cycle

The ability of GLI1 to act as a transcriptional regulator is often analysed using reporter constructs. This is mainly due to the lack of knowledge about the GLI-mediated transcriptional programme and the cellular specificity of many known GLI1 targets. In an attempt to avoid the usage of reporter constructs, we turned our attention to HPV genomes.

The rationale for these experiments was the following. First, we observed that both GLI1FL and GLI1ΔN were expressed in NHEKs and U2OS cells, two cell lines supporting HPV early transcription and replication [17]. Second, according to RNA-seq databases, *GLI1* is expressed at relatively high levels in normal cervix, the natural target tissue of many HPV types, and in cell lines derived from cervical cancer [21]. Therefore, it is likely that the co-occurrence of GLI1 proteins and HPV genomes also exists *in vivo*. Third, we observed several putative non-consensus GLI binding sites located in the noncoding region of the HPV18 genome. Here, we show that GLI1FL, but not GLI1ΔN, is capable of acting as a transcriptional repressor of HPV18 early promoters. While GLI1 is generally thought to act only as a transcriptional activator, several groups have also suggested that it might repress certain genes in a context dependent manner [30][31]. Although such overexpression experiments are often difficult to interpret, we believe that the significant difference between the repressor activities of GLI1FL and GLI1ΔN indicates that the effect is direct and not an experimental artefact. Currently, we are unable to pinpoint the exact reason for this differential activity, but we have ruled out the GLI1FL and GLI1ΔN differential DNA binding and regulation of the cell cycle. At least two additional explanations for this phenomenon come to mind. First, it is plausible to speculate that due to the close proximity of the GLI and E2 binding sites in the HPV18 upstream regulatory region, binding of GLI1FL to its cognate site sterically hinders the binding of E2, the main regulator of viral transcription, whereas the binding of GLI1ΔN does not have such an effect because of its smaller size or absence of binding partners interacting specifically with the N-terminal part of GLI1FL. Second, it is possible that the unique N-terminus of the GLI1FL protein harbours a transcriptional repression domain in itself.

Given that GLI1FL was able to repress early transcription of HPV18, we were interested in whether this protein also has an impact on the efficiency of viral genome replication. Two other HPV types were included in this analysis. HPV11 is a low-risk type infecting mucosal epithelium similar to HPV18. The HPV11 early promoter is similar to that of HPV18. HPV5 is a skin infecting type.

GLI1FL was able to inhibit the replication of the HPV11 and HPV18 genomes approximately 2-fold at early time points after transfection; later, the levels of inhibition decreased. This result was expected, as the expression of exogenous GLI1 declines over time due to cell proliferation, while viral genomes are sustained, replicating and segregating between daughter cells. GLI1ΔN also had a very modest negative effect on the replication of the HPV11 and HPV18 genomes, which could be explained by the fact that the E2 protein and E2 binding sites have a distinct function in the initiation of HPV replication, which is loading of the viral helicase E1 onto the origin of replication.

Surprisingly, we observed a very strong negative effect of both GLI1FL and GLI1ΔN on the replication of HPV5 at all time-points. As in the inhibition of HPV11 and HPV18 replication, the impact of GLI1FL was also more robust. Our bioinformatic analysis revealed that in contrast to HPV11 and HPV18, the predicted GLI1 binding site almost completely overlaps with an E2 binding site, and therefore, its occupation by GLI1FL and GLI1ΔN may have a direct impact on the initiation of HPV5 replication. Finally, not only localization but also a number of potential GLI binding sites in the particular viral genome may influence the level of inhibition. Further studies are needed to clarify the effect of GLI1 transcription factors on the life cycle of different HPV types.

## Supporting information

S1 Fig Linear regression analysis reveals a correlation between GLI1FL and GLI1ΔN mRNA levels. *GLI1FL* and *GLI1ΔN* mRNA levels were measured in thirteen cell lines using qPCR and normalized to *GAPDH* mRNA expression levels. Linear regression analysis was performed using GraphPad software.
(TIF)

S2 Fig Peptide corresponding to the N-terminus of GLI1ΔN can be identified from the immunocomplex precipitated from the 1321 N1 cells using trypsinolysis and mass spectrometry. MS chromatogram of the GLI1ΔN N-terminal peptide MSPSLGFPAQMNHQK in its 3+ and 2+ charge with oxidized methionine states **B.** MS/MS chromatogram of the respective peptides.
(TIF)

S3 Fig Sub-cellular localization of overexpressed GLI1Fl and GLI1ΔN proteins in HUH7 cells. Flag-tagged GLI1 isoforms (green signal) and nuclei (blue signal) were visualized using immunofluorescence microscopy. Majority of the GLI1 signal is localized into the nucleus, although there are cells, where the green signal can be seen also in the cytoplasm.
(TIF)

S4 Fig Quantification of the SB signals. U2OS cells were transfected with different HPV genomes and the GLI1FL or GLI1ΔN encoding constructs. Total DNA was extracted 2, 3 and 4 days post-transfection, digested with DpnI and other restriction enzymes to linearize the HPV genomes, and analysed using SB. The signals corresponding to the replicated HPV genomes were quantified using ImageQuant software and set as 100% in the samples transfected with the empty vector and incubated for 2 days. Data are presented as the mean of 3 independent experiments +/- SD (*—p < 0.05, **—p < 0.01, ***—p < 0.001).
(TIF)

S1 File Supplementary methods (related to S2 Fig). (DOCX)

S2 File Raw data images. (PDF)
