# Supplementary figures and images for: Differential phosphorylation determines the repressor and activator potencies of GLI1 proteins and their efficiency in modulating the HPV life cycle
Source: PLoS One. 2019 Nov 26;14(11):e0225775. doi: 10.1371/journal.pone.0225775 (PMC6879148; doi:10.1371/journal.pone.0225775)

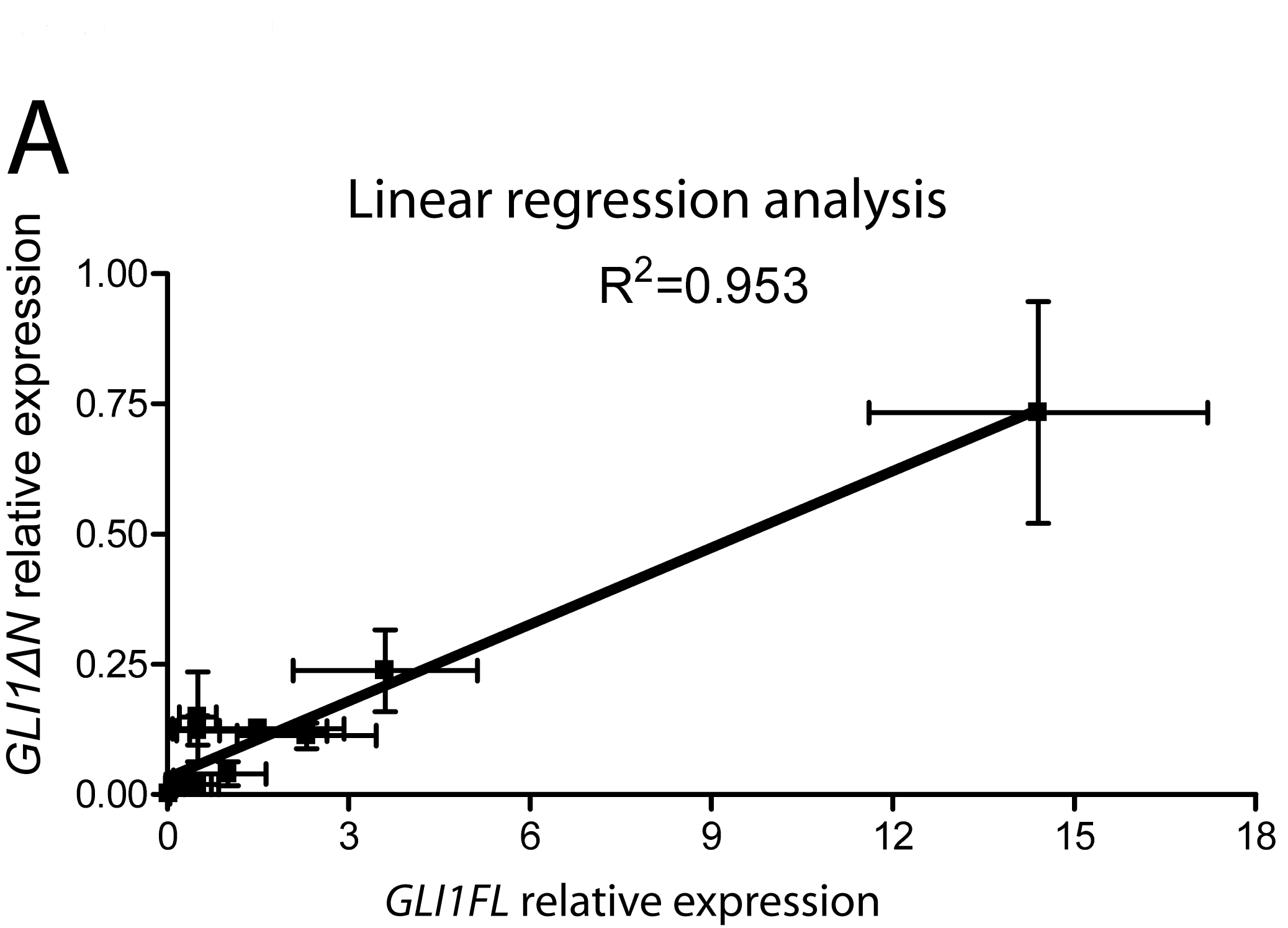

Supplement: S1 Fig — GLI1FL and GLI1ΔN mRNA levels were measured in thirteen cell lines using qPCR and normalized to GAPDH mRNA expression levels. Linear regression analysis was performed using GraphPad software. (TIF) [file pone.0225775.s001.tif]

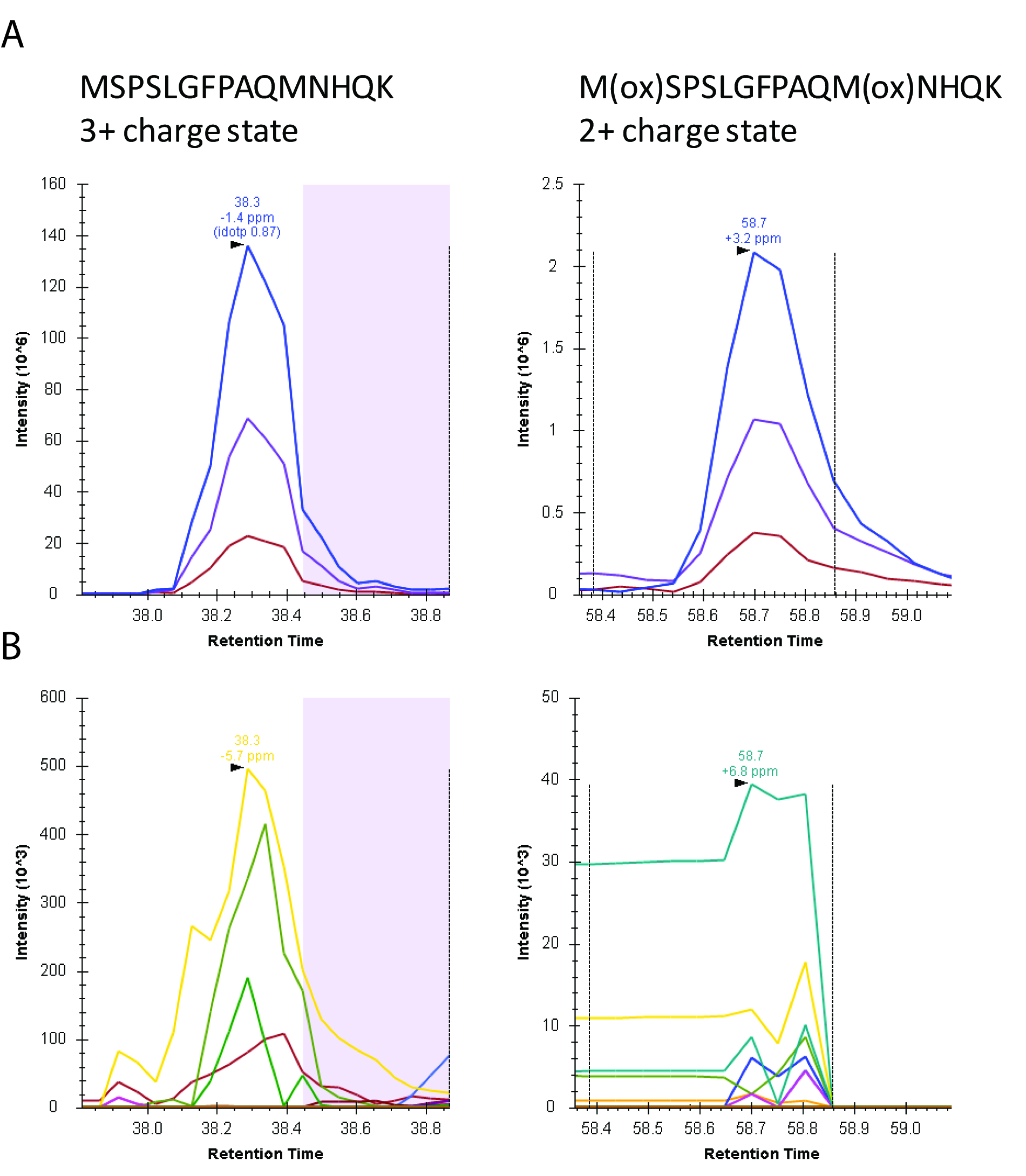

Supplement: S2 Fig — MS chromatogram of the GLI1ΔN N-terminal peptide MSPSLGFPAQMNHQK in its 3+ and 2+ charge with oxidized methionine states B. MS/MS chromatogram of the respective peptides. (TIF) [file pone.0225775.s002.tif]

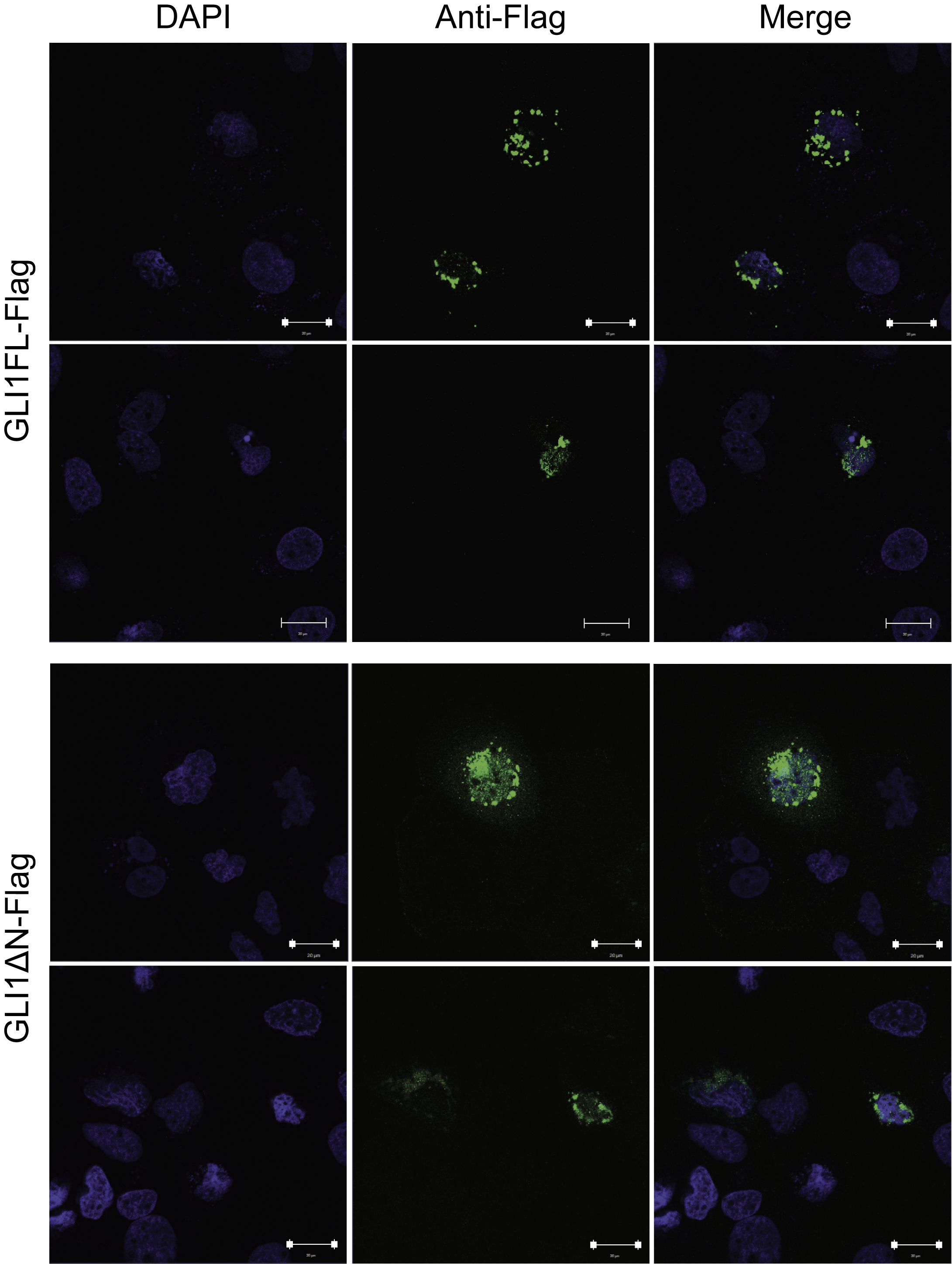

Supplement: S3 Fig — Flag-tagged GLI1 isoforms (green signal) and nuclei (blue signal) were visualized using immunofluorescence microscopy. Majority of the GLI1 signal is localized into the nucleus, although there are cells, where the green signal can be seen also in the cytoplasm. (TIF) [file pone.0225775.s003.tif]

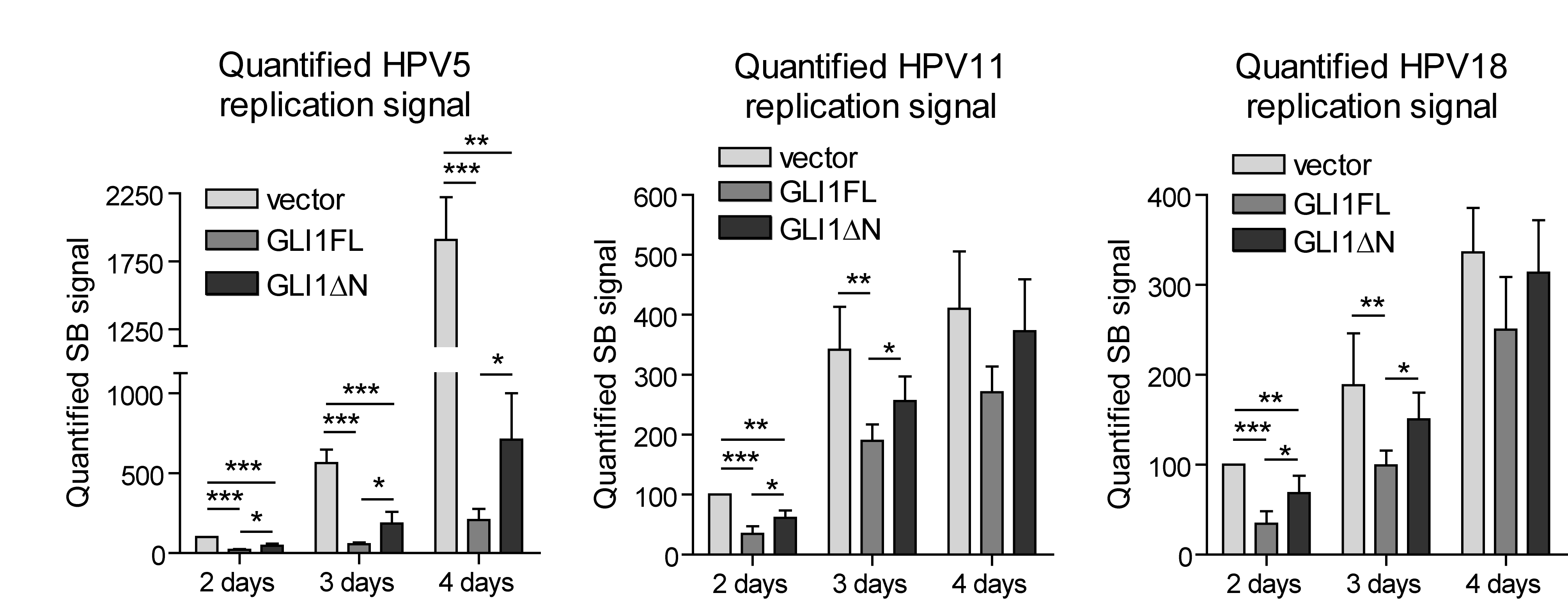

Supplement: S4 Fig — U2OS cells were transfected with different HPV genomes and the GLI1FL or GLI1ΔN encoding constructs. Total DNA was extracted 2, 3 and 4 days post-transfection, digested with DpnI and other restriction enzymes to linearize the HPV genomes, and analysed using SB. The signals corresponding to the replicated HPV genomes were quantified using ImageQuant software and set as 100% in the samples transfected with the empty vector and incubated for 2 days. Data are presented as the mean of 3 independent experiments +/- SD (*—p < 0.05, **—p < 0.01, ***—p < 0.001). (TIF) [file pone.0225775.s004.tif]

Figure 2B

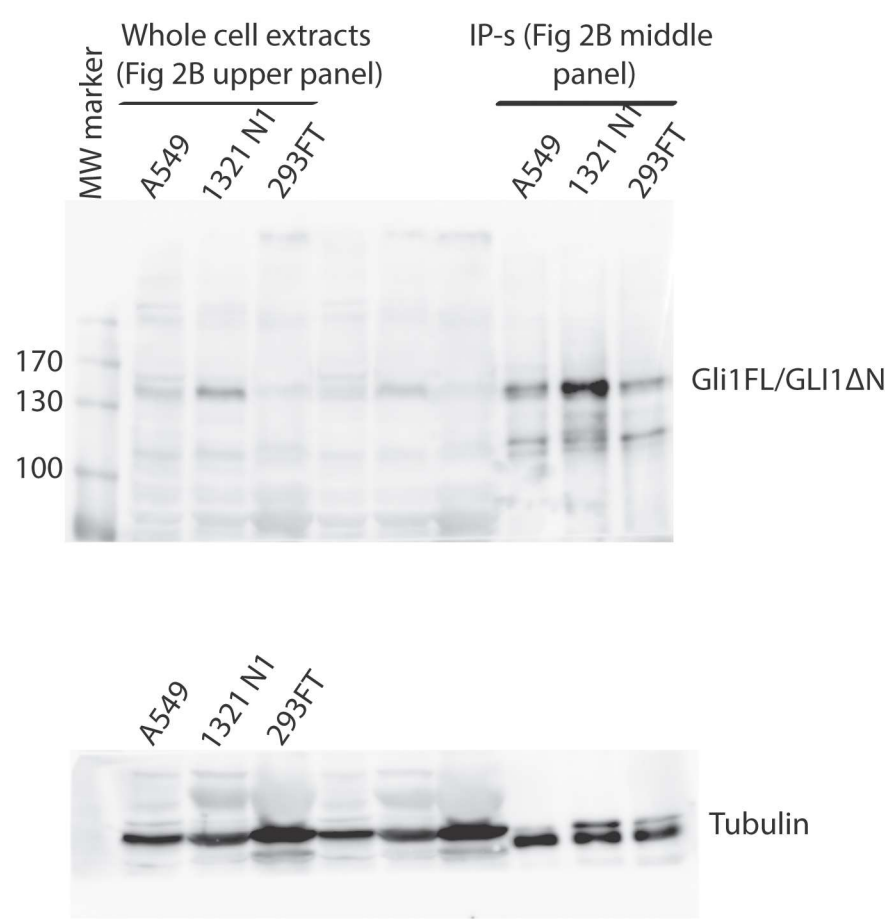

Figure 3B

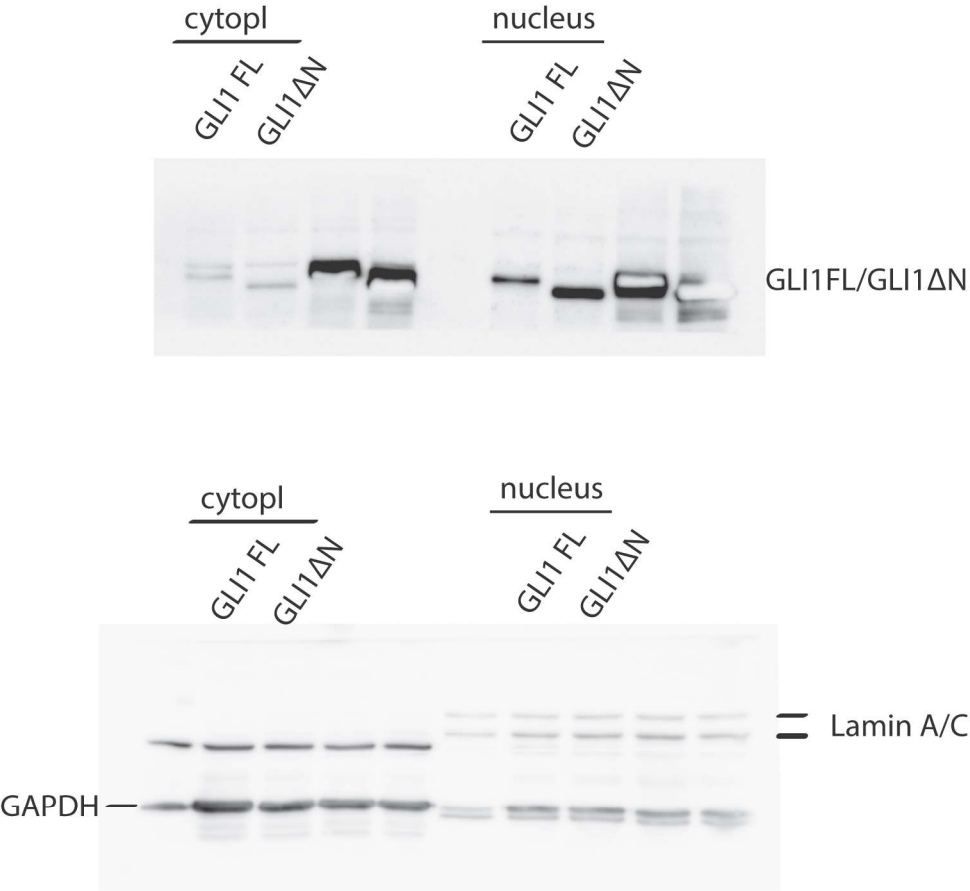

Figure 3C

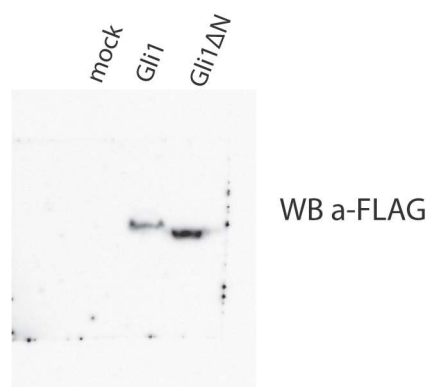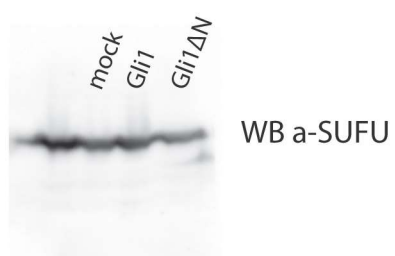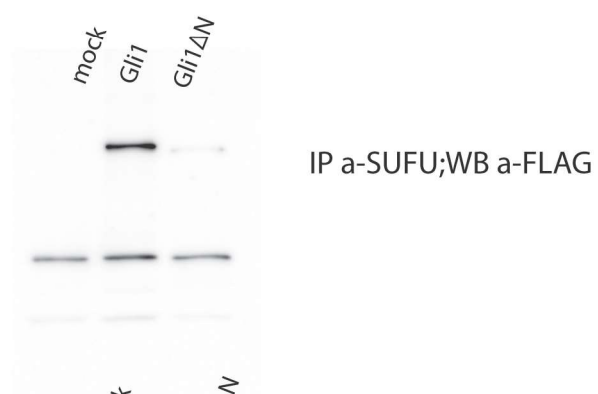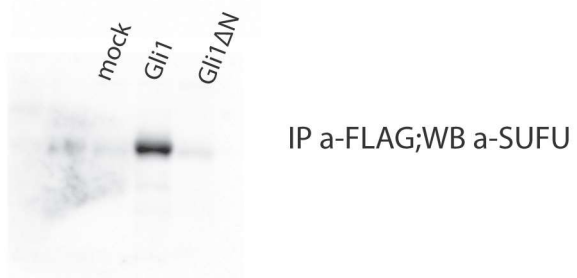

Figure 4B

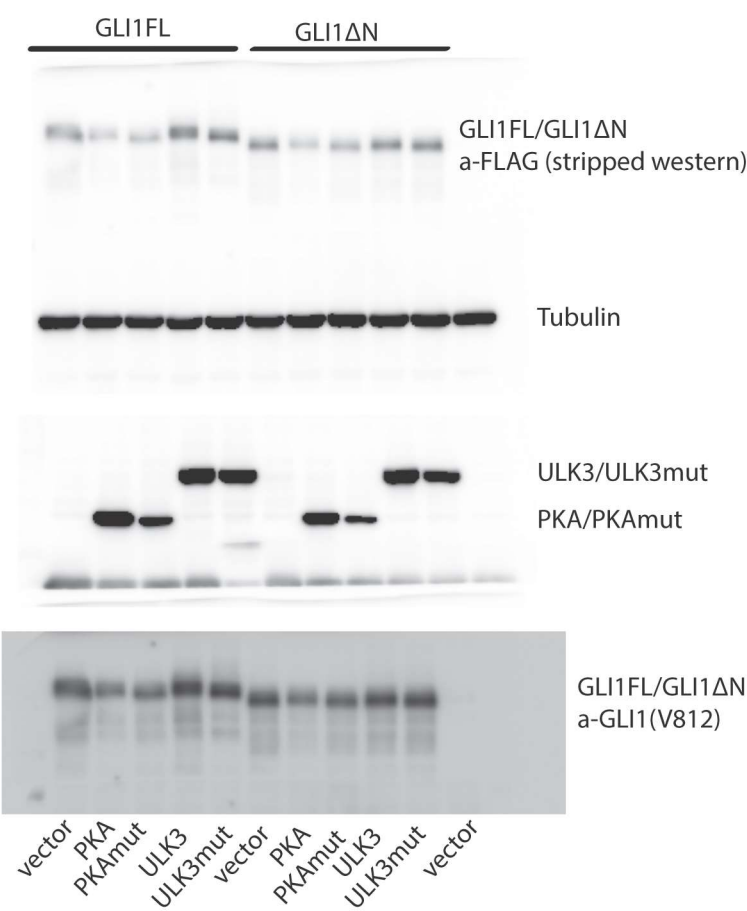

Figure 5A

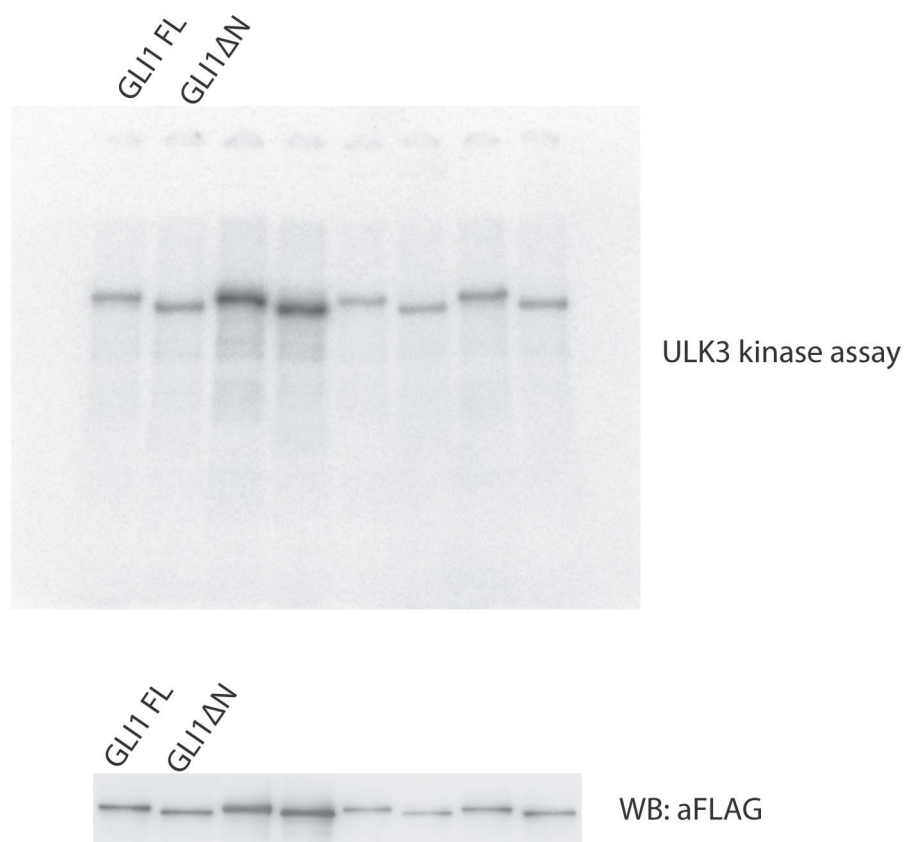

Figure 5B

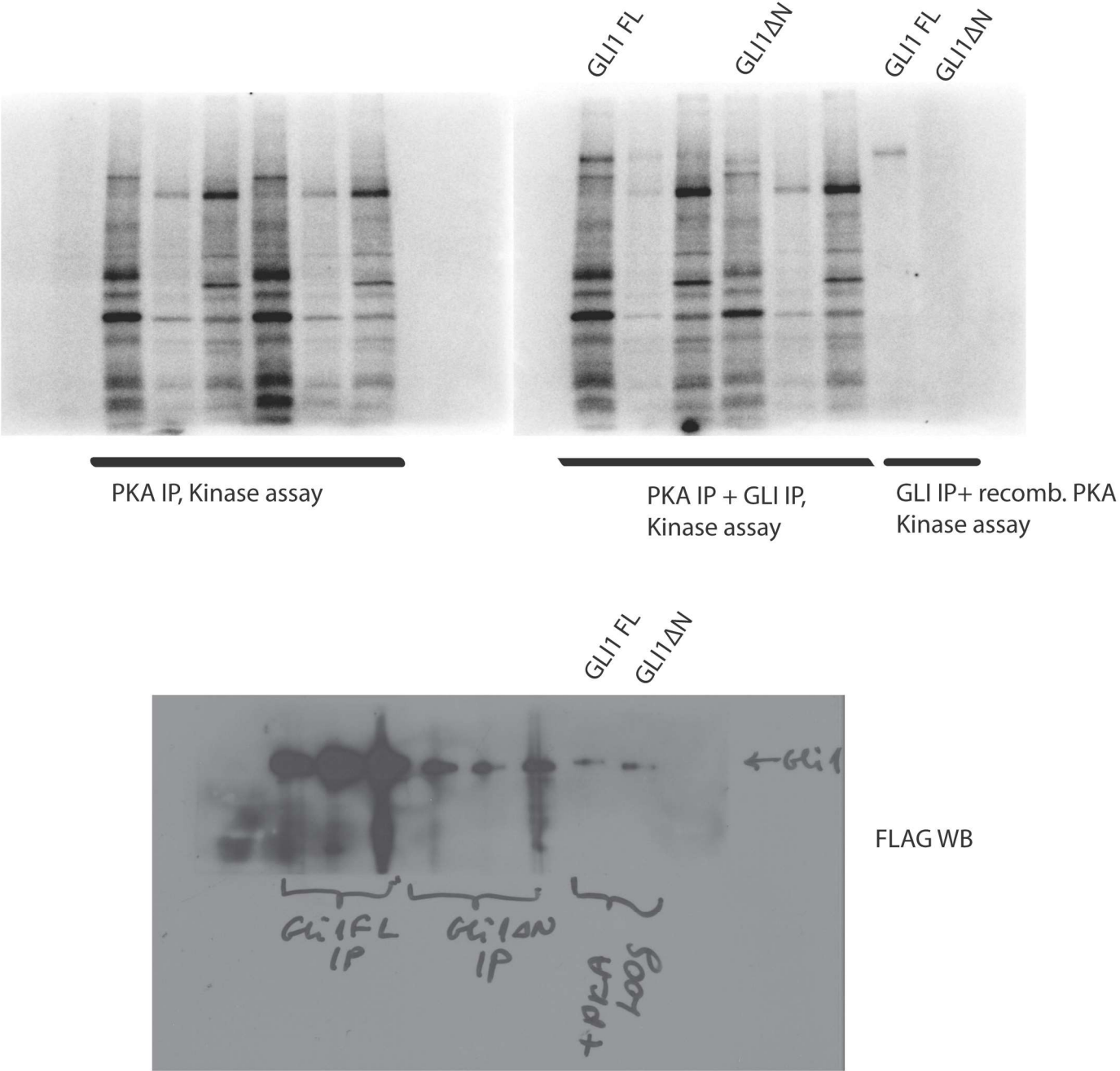

Figure 6B

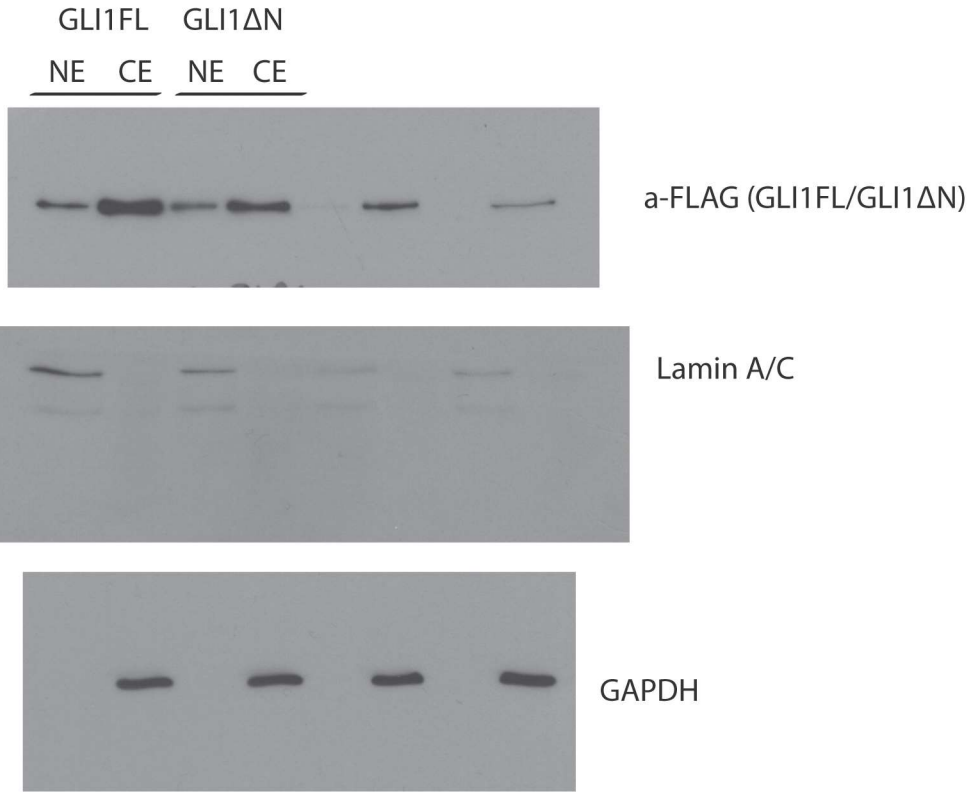

Figure 6C

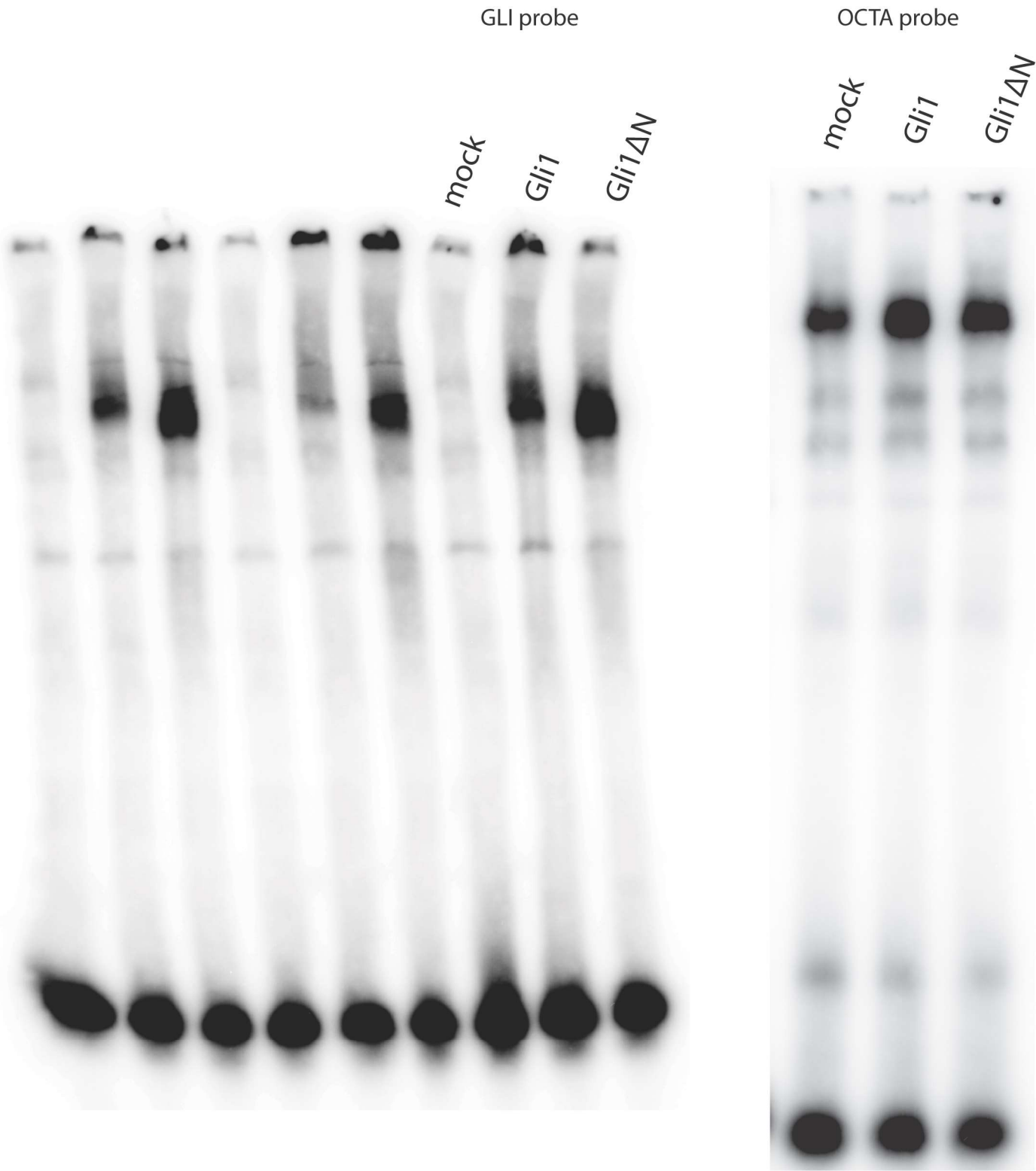

Figure 7A

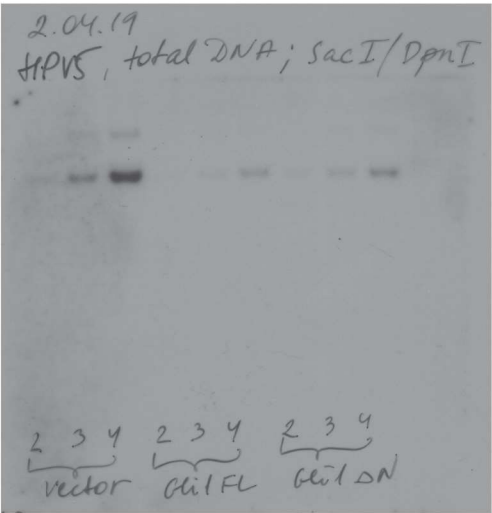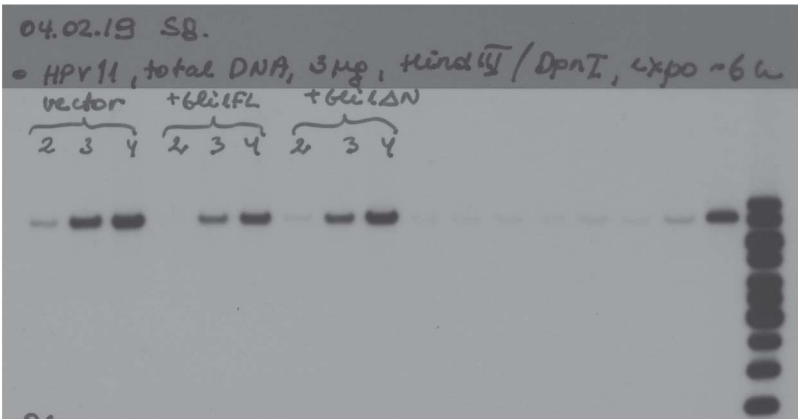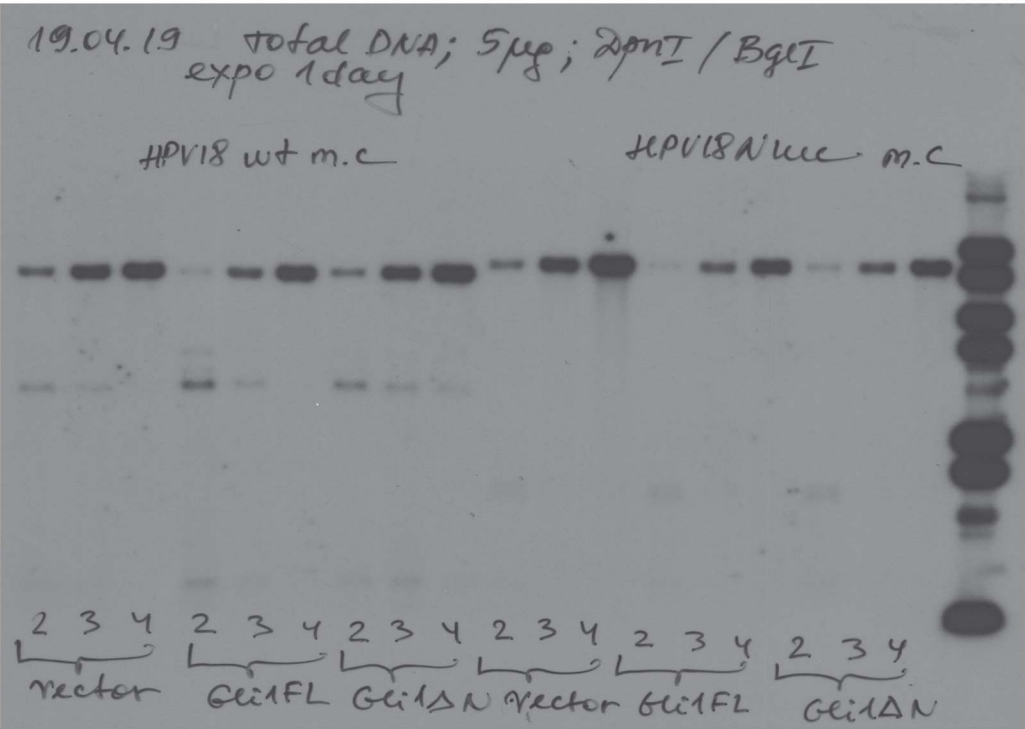

Supplement: S2 File — (PDF) [file pone.0225775.s006.pdf]
