# Supplementary material for: Differential phosphorylation determines the repressor and activator potencies of GLI1 proteins and their efficiency in modulating the HPV life cycle
Source: PLoS One. 2019 Nov 26;14(11):e0225775. doi: 10.1371/journal.pone.0225775 (PMC6879148; doi:10.1371/journal.pone.0225775)
Supplement: S1 File — (DOCX) [file pone.0225775.s005.docx]

**Supplementary methods (related to Figure S2)**

**MS analysis**

In-solution digestion of the immunocomplexes was performed by washing the beads in 100 µl of pre-elution buffer (50 mM Tris-HCl pH 8.5, 1 mM EGTA, 75 mM KCl), and subsequent elution with vortexing in 40 µl of 7 M urea / 2 M thiourea 100 mM ABC solution for 30 min. The supernatant was transferred to a new tube and the elution was repeated. Disulfide reduction and cysteine alkylation with 5 mM DTT and 10 mM chloroacetamide were carried out for 30 min each at room temperature (RT). Proteins were predigested with 400 ng of Lys-C (Wako Chemicals) for 4 h, diluted 5 times with 100 mM ABC and further digested with 400 ng of trypsin (Sigma-Aldrich) overnight at RT. Peptides were desalted with in-house made C18 StageTips.

Targeted nano-LC/MS/MS was performed by injecting the desalted peptides to an Ultimate 3000 RSLCnano system (Dionex) using a 0.3 x 5 mm trap-column (5 µm C18 particles, Dionex) and an in-house packed (3 µm C18, Dr Maisch) analytical 50 cm x 75 µm emitter-column (New Objective). Peptides were eluted at 250 nl/min with a 10-45% B 60 min gradient (buffer A: 0.1 % formic acid, buffer B: 80 % acetonitrile + 0.1 % formic acid) to a quadrupole-Orbitrap (Q Exactive Plus, Thermo Fisher Scientific) mass spectrometer using a nano-electrospray source (positive mode, spray voltage of 2.5 kV). The MS was operated in a targeted single ion monitoring data-dependent MS/MS mode (tSIM-ddMS2) by monitoring peptide precursor ions from GLI1 and triggering a fragmentation if specified peptide ions, as shown in Table 2, were observed within +/- 5 ppm. The MS and MS/MS isolation windows were 4.0 and 2.0 m/z, respectively. MS and MS/MS scans were performed at a resolution setting of 70 000 and 35 000 with ion target values set to 1e5 (injection time 120 ms). The normalized collision energy was set at 26.

Table. Expected peptide precursor ions from GLI1

| m/z, Th | Charge state, z | Peptide and modification(s) |
| --- | --- | --- |
| 865.903 | 2 | M[acetylation]SPSLGFPAQM[oxidation]NHQK |
| 857.906 | 2 | M[acetylation]SPSLGFPAQMNHQK |
| 558.269 | 3 | MSPSLGFPAQMNHQK |
| 836.900 | 2 | MSPSLGFPAQMNHQK |
| 1060.483 | 3 | C[carbamidomethylation]TSPGGSYGHLSIGTM[oxidation]SPSLGFPAQM[oxidation]NHQK |
| 1201.518 | 2 | M[acetylation]FNSM[oxidation]TPPPISSYGEPC[carbamidomethylation]C[carbamidomethylation]LR |
| 1193.520 | 2 | M[acetylation]FNSMTPPPISSYGEPC[carbamidomethylation]C[carbamidomethylation]LR |

Raw data were analysed with the Skyline software suite [1]. Extracted ion chromatograms were generated from precursors and fragments by integrating p- and y-ions across the RT-plane. Data were manually inspected for co-elution and mass accuracies.

**References**

1. MacLean, B., Tomazela, D. M., Shulman, N., Chambers, M., Finney, G. L., Frewen, B., Kern, R., Tabb, D. L., Liebler, D. C., and MacCoss, M. J. (2010) Skyline: An open source document editor for creating and analyzing targeted proteomics experiments. *Bioinformatics*. 10.1093/bioinformatics/btq054
